# Interactions of Pleckstrin Homology Domains with Membranes: Adding Back the Bilayer via High-Throughput Molecular Dynamics

**DOI:** 10.1016/j.str.2016.06.002

**Published:** 2016-08-02

**Authors:** Eiji Yamamoto, Antreas C. Kalli, Kenji Yasuoka, Mark S.P. Sansom

**Affiliations:** 1 Department of Mechanical Engineering, Keio University, 3-14-1 Hiyoshi, Kohoku-ku, Yokohama-shi, Kanagawa-ken 223-8522, Japan; 2 Department of Biochemistry, University of Oxford, South Parks Road, Oxford OX1 3QU, UK

## Abstract

A molecular simulation pipeline for determining the mode of interaction of pleckstrin homology (PH) domains with phosphatidylinositol phosphate (PIP)-containing lipid bilayers is presented. We evaluate our methodology for the GRP1 PH domain via comparison with structural and biophysical data. Coarse-grained simulations yield a 2D density landscape for PH/membrane interactions alongside residue contact profiles. Predictions of the membrane localization and interactions of 13 PH domains reveal canonical, non-canonical, and dual PIP-binding sites on the proteins. Thus, the PH domains associate with the PIP molecules in the membrane via a highly positively charged loop. Some PH domains exhibit modes of interaction with PIP-containing membranes additional to this canonical binding mode. All 13 PH domains cause a degree of local clustering of PIP molecules upon binding to the membrane. This provides a global picture of PH domain interactions with membranes. The high-throughput approach could be extended to other families of peripheral membrane proteins.

## Highlights

• Interactions of 13 different PH domains with PIP-containing membranes are simulated
• PH domains associate with PIPs in membranes via a positively charged loop
• Binding of PH domains causes clustering of PIP molecules within membranes
• Simulations reveal canonical and alternative binding modes of PIPs to PH domains

## Graphical Abstract

**Figure:**
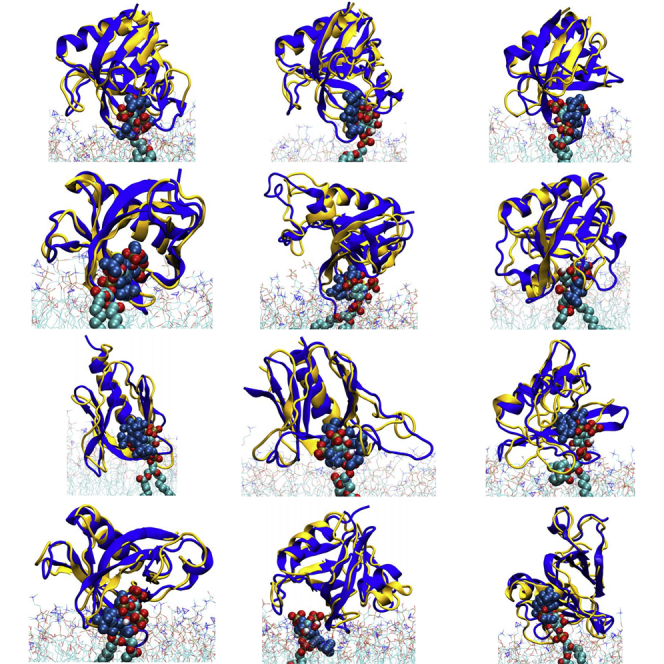

## Teaser

Using a molecular simulation pipeline Yamamoto et al. reveal the interactions of 13 PH domains with PIP-containing lipid bilayers. Some PH domains exhibit modes of interaction with PIP-containing membranes additional to the canonical binding mode. All the PH domains cause a degree of local clustering of PIP molecules upon binding to the membrane.

## Introduction

The association of peripheral membrane proteins (PMPs) with cell membranes is crucial for many cellular functions, including cell signaling and trafficking (Cho and Stahelin, 2005). This association is often mediated by lipid-binding modules, e.g., the pleckstrin homology (PH) domain found in many PMPs (Lemmon, 2008). Determining the interactions of such domains with the membrane at the molecular level is central to our understanding of the function of PMPs. PMPs interact with the surface of cell membranes via a mixture of specific and non-specific interactions, which sometimes include contributions from covalently attached lipid anchors (Hancock, 2003). Association of PMPs with cell membranes is often controlled by binding to specific lipids, e.g., to phosphatidylinositol phosphates (PIPs) present in cell membranes (Balla, 2005, Stahelin et al., 2014).

The number of PMPs structures has increased significantly during recent years. For example, there are currently ∼150 structures of PH domains deposited in the PDB. Although numerous PMP structures have been determined, both by X-ray crystallography and by nuclear magnetic resonance (NMR), only rarely do such structures reveal directly the nature of their interactions with membranes. Indeed, structures of PMPs often do not contain bound lipid molecules. Even when there is a lipid molecule bound in a crystal structure, it is often simply the head group of the lipid that is bound to the PMP. This provides a radically simplified model of the in vivo environment in which PMPs function, and provides only indirect indications as to their exact position and orientation on a cell membrane. Using such structural data, it remains challenging to understand the mechanistic details of their association with membranes and of their interactions with lipid molecules that may be also coupled with conformational changes within the protein and penetration of parts of the protein into the bilayer. We, therefore, need to characterize the modes of interaction of PMPs with their target cell membranes in order to understand the relationship between their molecular structure and biological function. Biophysical studies (e.g., NMR and single-molecule fluorescence microscopy; Knight et al., 2010, Kutateladze and Overduin, 2001, Stahelin et al., 2014) can provide some information, but such detailed characterization is not available for the majority of PMPs. This is especially likely to be the case as higher-throughput experimental approaches are used to explore the interactions between membranes and PMPs (Best, 2014, Vonkova et al., 2015).

Molecular dynamics simulations provide a computational approach to characterize the interactions of membrane proteins with their lipid bilayer environment (Stansfeld and Sansom, 2011b), and in particular to study the interactions of PMPs with model membranes (Kalli and Sansom, 2014, Vermaas et al., 2015). Recently, high-throughput molecular dynamics simulations have been used to study, e.g., the oligomerization of TM helices (Wassenaar et al., 2015), the association of phosphatase and tensin homolog with model membranes (Kalli et al., 2014), or anomalous dynamics of DAPP1 PH domain on model membranes (Yamamoto et al., 2015). Comparisons with experiments have shown that these simulations are in good agreement with available experimental data. In this study we present a high-throughput molecular dynamics simulation protocol that allows us to study the interaction of PMPs with model membranes. This approach was applied to a family of PMPs for which we have many structural and functional data, i.e., the PH domains.

PH domains are an important class of membrane recognition domains that bind to specific lipids (PIPs) in cell membranes. Many structures of PH domains are known, some (ca. 13) with bound inositol-phosphates (IPs, i.e., PIP headgroups). Each PH domain consists of ∼120 residues with an antiparallel β sheet architecture followed by one or two amphipathic α helices (Stahelin et al., 2014). The majority of PH domains have a KXn(K/R)XR motif in the loop connecting strands β1 and β2. This positively charged sequence was shown to regulate the contacts of the PH domains with different types of PIP molecules (Ferguson et al., 2000, Moravcevic et al., 2012). There are, however, a number of PH domains that do not have this consensus sequence, e.g., the β-spectrin and ArhGap9 PH domains (Ceccarelli et al., 2007, Hyvönen et al., 1995, Moravcevic et al., 2012). For these PH domains it was shown that the binding of PIP lipids occurs on the opposite face of the β1/β2 strands. Interestingly, recent studies (Jian et al., 2015, Vonkova et al., 2015) suggest that the nature of the interactions of PH domains with the target membrane may be more complex than simple recognition of a single lipid (PIP) headgroup. A recent structure of the ASAP1 PH domain suggested that PIP may bind to both a canonical site (similar to that found in those PH domains that have the KXn(K/R)XR motif) and to a non-canonical site (similar to the PH domains that do not have the KXn(K/R)XR motif) (Jian et al., 2015). In the PDB there are structures for all three types of PH domains, and thus in our study we have examined the binding to model membranes of all three different types of PH domains. In particular, we have studied GRP1, ARNO, PLC-δ1, DAPP1, PDK1, PEPP1, PKB/Akt, C-PH, Kindlin-2, and Btk PH domains (Baraldi et al., 1999, Cronin et al., 2004, Ferguson et al., 1995, Ferguson et al., 2000, Jackson et al., 2007, Komander et al., 2004, Lietzke et al., 2000, Liu et al., 2011, Milburn et al., 2003) that do have the KXn(K/R)XR motif (canonical PIP-binding site); ArhGAP9 and β-spectrin PH domains (Ceccarelli et al., 2007, Hyvönen et al., 1995) that do not have the KXn(K/R)XR motif (non-canonical PIP-binding site), and the ASAP1 PH (Jian et al., 2015) domain that is proposed to have both canonical and non-canonical PIP-binding sites.

Here, we present a computational pipeline for studying the interactions of PH domains with PIP-containing membranes. We evaluate this method for the canonical PH domain of GRP1. We derive a 2D density landscape for the protein/membrane interaction alongside residue contact profiles that fingerprint the protein/PIP interactions. We investigate the localization on the surface of a model membrane of 13 different PH domains for which there are structures for the PH/(P)IP complex in the PDB. Our results demonstrate that some PH domains are predicted to have modes of interaction with PIP-containing membranes additional to the canonical binding mode. These studies provide a global picture of PH domain interactions with membranes, and exemplify high-throughput molecular dynamics simulations as a more general protocol for exploring PMP/membrane interactions.

## Results

### GRP1 PH Domain: a Canonical PH Domain to Develop and Evaluate the Method

**Figure 1:**
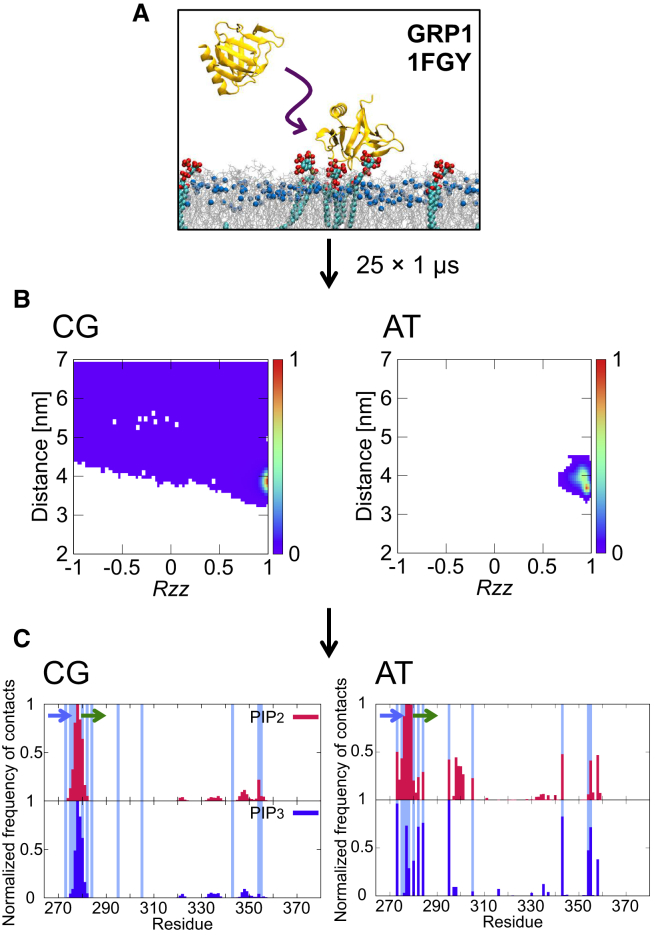
The PH Domain/Bilayer Simulation Pipeline (A) Snapshot of a selected simulation demonstrating the localization of the GRP1 PH domain to the lipid bilayer. The GRP1 PH domain is shown in yellow. PIP_3_ molecules are shown in green/red/bronze, and the POPC and POPS lipids are shown as silver lines (phosphorus atoms, blue). See also Figure S1. (B) Normalized density map of the GRP1 PH domain (*zz* component of rotational matrix versus distance). (C) Normalized average number of contacts between the GRP1 PH domain protein and PIPs shown for the 25 × 1 μs CG-MD simulations and for the 2 × 1 μs AT-MD simulations (see also Figure S1). The light blue colors represent the experimental contacts observed in the crystal structure. For normalization, the number of contacts of a residue with a lipid type was divided by the largest number of contacts that the same lipid type made with any residue in the protein. This means that the residue with the most frequent contacts will have the value of 1 and the residue with no contacts with a lipid type will have the value of 0. The position of the β1 and β2 strands is shown by blue and green arrows, respectively. Contacts were defined using cut-off distances of 0.7 and 0.4 nm, respectively for CG-MD and AT-MD simulations. The same analysis for the atomistic simulations of the PLC-δ1 PH and the β-spectrin PH domains is shown in Figure S2.

Our simulation pipeline (Figure 1) was first tested using the GRP1 PH domain (see Table 1) for which combined biophysical data and atomistic molecular dynamics (AT-MD) simulations have demonstrated the preferred (i.e., canonical) mode of interaction of the PH domain with a PIP_3_ molecule in a phospholipid bilayer (Lumb et al., 2011). At the start of each coarse-grained molecular dynamics (CG-MD) simulation, the PH domain was placed in a simulation box at a distance of 7 nm away from a preformed PC/PS/PIP_2_/PIP_3_ (73%/20%/5%/2%) lipid bilayer. An ensemble of 25 repeat simulations was performed, with each simulation of duration 1 μs and starting from different initial orientations of the PH domain relative to the bilayer. During the simulations, the PH domain diffuses in the aqueous environment before encountering and forming a complex with the PIP-containing lipid bilayer (Figure 1A).

To quantify the binding of the GRP1 PH domain to the membrane, the progress of each simulation was tracked in terms of the distance from and orientation of the PH domain relative to the lipid bilayer. Merging these data across the whole ensemble allows one to construct a 2D density map describing the interaction of a PH domain with a model membrane (see Experimental Procedures for details). The resultant density map may be described in terms of the number and depth of the density minima. For the GRP1 PH domain there is a single maximum, corresponding to a single preferred orientation of the protein relative to the membrane. This orientation corresponds well with that previously determined by Lai et al. (2013) using atomistic simulations, and is similar to the GRP1/membrane complex derived by Lumb et al. (2011) combining NMR observations and atomistic simulations. We have confirmed the convergence of the density map calculations using different numbers of repeat simulations in the ensemble for three different PH domain systems (see Figure S3). By way of comparison, we note that experimental measurements of the dissociation constant (K_d_) of GRP1 PH with PtdIns(3,4,5)P_3_ or Ins(3,4,5)P_3_ molecules in solution yielded values of 50 nM (corresponding to ca. −10 kcal/mol) (Klarlund et al., 2000) and ∼30 nM (Kavran et al., 1998), respectively. A K_d_ of 50 nM for the GRP1 PH domain binding to PIP_3_ in an anionic bilayer (Corbin et al., 2004) was estimated using a fluorescence resonance energy transfer competitive binding assay in which IP_6_ molecules were used to dissociate GRP1 PH from bilayers containing PIP_3_ molecules and other anionic lipids (i.e., phosphatidic acid).

We have examined the main contacts of the PIP molecules with the bound PH domain, averaged over the ensemble of simulations. We have analyzed these both for the ensemble of CG simulations, and also for the atomistic simulations launched from the preferred CG orientation. Protein/lipid contacts for the GRP1 PH domain with PIP_3_ and with PIP_2_ are shown in Figure 1C. The main contacts are, as anticipated, with the β1/β2 loop. Comparison of our results with the crystallographically observed contacts for bound Ins(1,3,4,5)P_4_ shows good agreement. The β6/β7 loop region also makes some contacts with the PIP molecules. Interestingly, in an NMR study (Lumb et al., 2011) of a PIP_3_-bound GRP1 PH domain in dodecylphosphocholine micelles, the protein amide resonances changed not only for the β1/β2 loop residues (around residue 280) but also for residues on the β6/β7 loop (around residue 350), both of which loops observed in the PIP_3_ contact analysis from the simulation ensemble. This is also consistent with the contacts seen in atomistic simulations of GRP1 modeled as bound to the head group of PIP_3_ in a lipid bilayer (Lumb et al., 2011). The main residues that interact with the PIP_2_ and PIP_3_ molecules are residues 273, 277, 278, 279, and 343. A recent study by Lai et al. (2013). also suggests that the interactions of the β1/β2 loop, and in particular residues R277 and K279, are important for the PH/PS lipid interactions. However, in our study we did not observe significant penetration of GRP1 V278 into the membrane, i.e., below the plane of phosphate in lipids (see Figure S1). In addition, the PIP-binding site was not flexible in our AT-MD simulations in good agreement with previous simulations of GRP1 bound to PIP_3_ (Lai et al., 2013) (see Figure S1). A similar orientation of the GRP1 PH domain relative to the bilayer was also observed in an electron paramagnetic resonance study (Chen et al., 2012). We are therefore confident that the results of the CG-MD protocol for the GRP1 PH/PIP interactions agree well with both experimental measurements and more detailed simulations by AT-MD. However, approximations implicit in the CG-MD simulations make it rather more difficult to analyze in detail the specificity of the PH domains for different PIP species. During the extended AT-MD simulations, the PIP lipid interactions with the PH domains were generally retained, with the exception of the PH domain of β-spectrin. This suggests that currently the AT-MD simulations remain too short to direct analysis of the specificity for different species of PIPs. We note that calculations of mean force potentials may allow us to study the specificity of interactions of PIP molecules with PH domains (Naughton et al., 2016), although this approach is currently only feasible for CG simulation models.

### A Comparison of 13 PH Domains

PH domains are a structurally conserved family of proteins, although there is a significant degree of sequence variation within the family. Using our high-throughput pipeline, we have extended our studies to multiple members of the PH family of proteins. Thus, we have selected 13 PH domains from the PDB databank for which there are experimentally derived PH/PIP headgroup structures (Baraldi et al., 1999, Ceccarelli et al., 2007, Cronin et al., 2004, Ferguson et al., 1995, Ferguson et al., 2000, Hyvönen et al., 1995, Jackson et al., 2007, Jian et al., 2015, Komander et al., 2004, Lietzke et al., 2000, Liu et al., 2011, Milburn et al., 2003) (i.e., for 12 complexes plus GRP1; see Table 1). Note that in this set of PH domains there are ten PH domains with canonical PIP-binding sites, two PH domains with non-canonical PIP sites, and one that exhibits two binding sites. Simulations were set up for all PH domains in the same manner as for GRP1 in order to study their interactions with and orientation relative to a model PIP-containing lipid bilayer.

**Figure 2:**
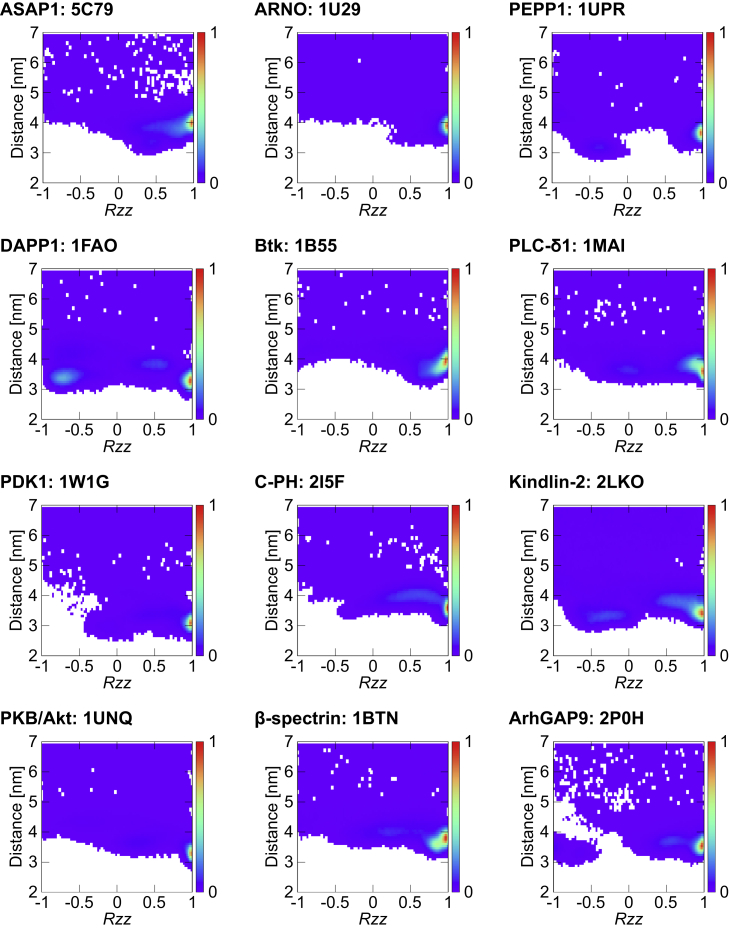
Normalized Density Maps for the 12 PH Domains, Other Than that of GRP1 For the density map of GRP1, see Figure 1C. The normalized density maps are shown as the *zz* component of the rotational matrix versus the *z* component of the distance between centers of mass of the protein and the bilayer. See also Figure S3 for convergence analysis and Figure S1 for the analysis of the orientation of the mutated form of the DAPP1 PH domain relative to the bilayer.

**Figure 3:**
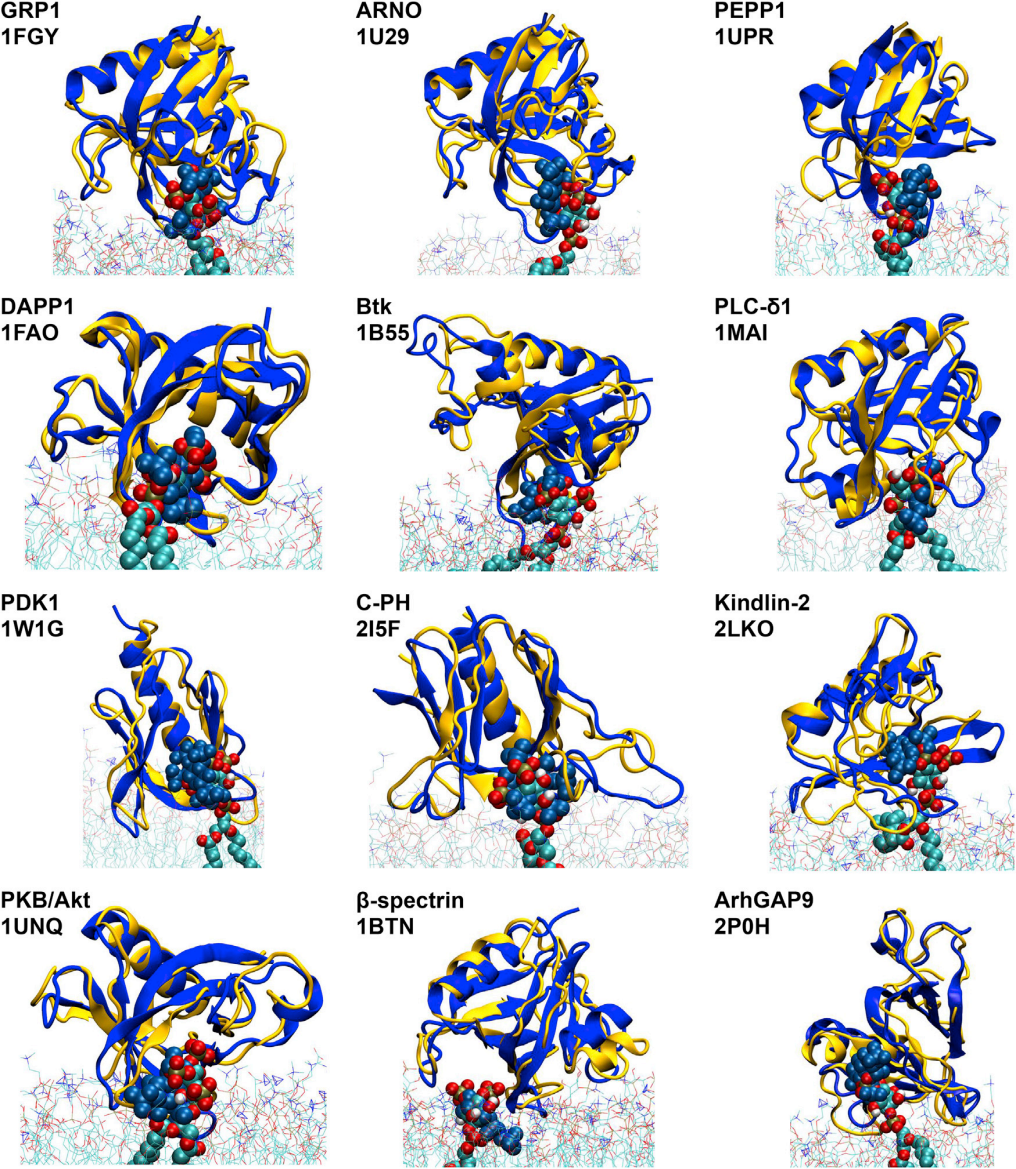
PH/PIP Complexes Alignment of the PH/PIP complexes derived from our simulation approach (with PH domains in yellow and PIP molecules in cyan/red/bronze/silver) with the corresponding crystal structures (PH domains and PIP both in blue). Note that PIPs in the simulation snapshots are located at approximately the same sites on the PH domains as in the crystal structures. These complexes were obtained from the maxima in the density maps shown in Figure 2. See also Figure S7.

Calculation of the density landscapes suggests that all of the PH domains adopt a preferred orientation (i.e., a global maximum) relative to the membrane, such that in this preferred orientation each PH domains has a bound PIP lipid at the binding sites suggested by the crystal/NMR structures of the PH/InsP complexes (Figure 2). Indeed comparison of the preferred orientation from each simulation (i.e., the PH/bilayer complex corresponding to the global maximum in the density landscape) with the experimental structures demonstrated that the PH/PIP complexes derived from our study are very similar to the complexes deposited in the PDB (Figure 3). Interestingly, in 83% of the final membrane complexes obtained by all our simulations (i.e., with 13 PH domains), a PIP molecule binds to the same binding site suggested by the PH/InsP complexes obtained by NMR or X-ray crystallography (see Table 1). We note that in the case of PLC-δ1 we also observe strong interaction of the PIP lipid(s) with the β3/β4 loop that is located next to the canonical β1/β2 loop. Strikingly, for β-spectrirn and ArhGAP9 domains, we observe the binding of the PIP lipid molecule on the opposite side of the β1/β2 loop (i.e., at a non-canonical PH site) as expected from the structural data. This is due to the fact that these PH domains lack a KXn(K/R)XR motif which is found in other PH domains (Ceccarelli et al., 2007, Moravcevic et al., 2012).

Closer examination reveals that some PH domains have more complicated density landscapes than others, despite the fact that for all of them the global maximum corresponds to a PH/bilayer complex in the canonical (i.e., preferred) orientation suggested by the experimental structures. For approximately 80% of the time (averaged across all simulation systems), the protein adopted a preferred orientation relative to the bilayer. In the remainder of the simulations, the PH domain adopted a perturbed orientation relative to the bilayer, but, in some of these simulations, a PIP molecule was still able to bind to the proposed PIP-binding site (see Table 1 for more details). In the simulations of GRP1, ARNO, Btk, PDK1, β-spectrin, and ArhGap9 PH domains, the PH/PIP complex was formed in the preferred orientation relative to the bilayer for more than 80% of the final complexes. In particular, GRP1 and ARNO exhibited only the canonical binding mode. The other PH domains had secondary binding modes. In these secondary binding modes, either the orientation of the PH domain changed slightly while retaining a PIP molecule at the canonical binding site or the PH domain interacted with the bilayer via a different positively charged region on the protein. In particular, in the simulations of the PEPP1, DAPP1, PLC-δ1, C-PH, Kindlin-2, PKB/Akt, and ASAP1, more than 60% of the final complexes were in the preferred orientation and had a bound PIP at the experimentally suggested binding sites. For the rest of these simulations, these PH domains were able to adopt alternative orientations relative to the membrane. However, as discussed above, in some of these cases, the PIP molecule was able to bind to the proposed PIP-binding site. Although such secondary binding modes of PH domains have not been previously discussed in detail, these modes may correspond to more transient modes of interactions before the PH domain adopts a canonical orientation. Consequently, the secondary modes may help to mediate the initial encounter between the PH domain and the membrane. However, we note that we used isolated PH domains in our simulations. The secondary binding modes may be sensitive to the presence or absence of the other domains given that PH domains are usually part of larger multi-domain structures.

**Figure 4:**
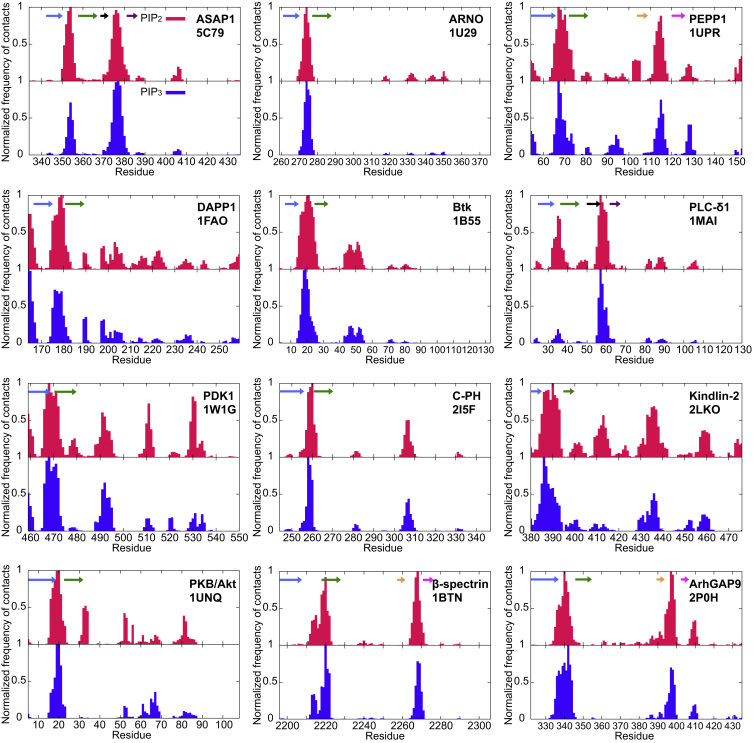
Normalized Average Number of Contacts between the PH Domains and PIPs Contacts were calculated using the whole ensemble (25 × 1 μs CG-MD simulations). For normalization, the number of contacts of a residue with a lipid type was divided by the largest number of contacts that the same lipid type made with any residue in the PH domain. The positions of the β1 to β6 stands are shown by blue, green, black, purple, orange, and pink arrows, respectively. See also Figures S5 and S6.

Analysis of the contacts between the PH domains and the PIP_2_ or PIP_3_ molecules suggests that all PH domains associate with the membrane via the positively charged loop connecting the β1 and β2 strands, in either a canonical or a non-canonical fashion (see Discussion above; Figure 4). Interestingly, the PH domains for which we have observed secondary binding modes, e.g., the Kindlin-2 PH domain exhibits additional contacts with other positively charged regions of the proteins. Calculation of the radial distribution functions for all the PH domains suggests that there is also a degree of clustering around the PH domain for both PIP_2_ and PIP_3_ lipids (see Figure S4). Clustering of PIP molecules has also been observed experimentally (Picas et al., 2014). Using CG-MD simulations, the fluctuation of the cluster size of PIPs around a PH domain was examined and found to exhibit 1/*f* noise (Yamamoto et al., 2015). This clustering also contributes to the additional PIP/protein contacts. In vivo studies of PDK1 (Lucas and Cho, 2011) and PKB/Akt (Huang et al., 2011) PH domains suggest that the interactions of the β1/β2 loop are important for PH/PS lipid interactions. These suggested interactions are also observed in our simulations (see Figures S5 and S6).

### Non-Canonical PIP Interactions as Exemplified by the PH Domain of ASAP1

**Figure 5:**
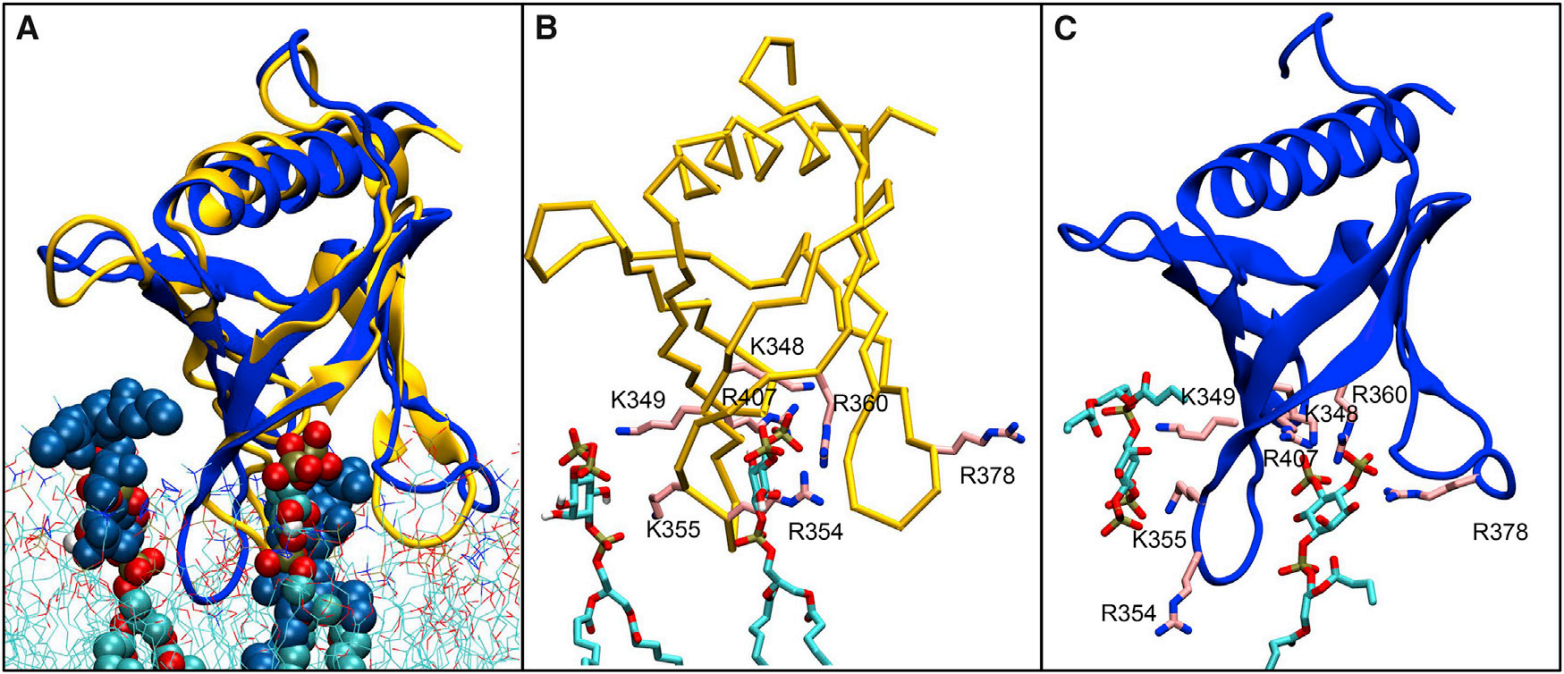
Binding of PIP Molecules to Both Canonical and Non-canonical Sites on the ASAP1 PH Domain (A–C) A simulation snapshot and the crystal structure are compared in (A) (the same format as in Figure 3 for other PH domains). (B) A snapshot of the PH/PIP_2_ complex derived from our CG simulations and then converted to an atomistic model, with the PH domain in yellow and the two bound PIP_2_ molecules (in cyan/red/bronze). (C) The crystal structure (PDB: 5C79) with the PH domain in blue and the two bound dibutyryl PIP molecules (in cyan/red/bronze/silver). See also Figure S2.

Recently, a crystal structure of the ASAP1 PH domain (PDB: 5C79) was determined in which the authors identified an “atypical” (A) binding site in addition to the “canonical” (C) PIP-binding site (Jian et al., 2015). The presence of an additional site on a PH domain may have regulatory and functional roles. Interestingly, analysis of our simulation with the ASAP1 PH domain also revealed that PIP lipids interacted with both the canonical and the atypical sites suggested by the crystal structure (Jian et al., 2015) (see Figure 5). A detailed atomistic simulation of the ASAP1 PH domain confirmed that both binding sites predicted by CG-MD simulations provided stable PH/PIP interactions (see Figure S2). However, inspection of the ASAP1 PH structure suggests that in the crystal the dibutyryl PIP_2_ molecule may have adopted an upside down orientation at the A site, as the (short C_4_) tails would point away from a bilayer, whereas in our simulations the PIP_2_ molecules at both the A and C sites have their alkyl tails pointing toward the membrane.

Binding of PIP molecules to atypical (i.e., non-canonical) lipid-binding sites has also been suggested for Sim1, Tiam, β-spectrirn, and ArhGap9 PH domains (Anand et al., 2012, Ceccarelli et al., 2007, Hyvönen et al., 1995, Moravcevic et al., 2012). These PH domains have the potential for cooperative binding of PIP molecules to canonical and non-canonical sites. There is also an in vivo study that suggests the existence of two lipid-binding sites of PKB/Akt PH domain (Huang et al., 2011). Overall, we observed binding of PIP molecules to both canonical and non-canonical sites for the PKB/Akt, β-spectrirn, and ArhGap9 PH domains (see Figure S7). Interestingly, in our AT-MD simulation of the β-spectrin PH domain, we observed dissociation of PIP_2_ from the non-canonical binding site (see Figure S2). After dissociation, the orientation of the PH domain switched to a different state, corresponding to the secondary orientation seen in the CG-MD simulations. This suggests that the binding via the non-canonical site is important for maintaining the preferable orientation of the PH domain on the membrane surface.

### Conservation of the Interactions with PIP Lipids

**Figure 6:**
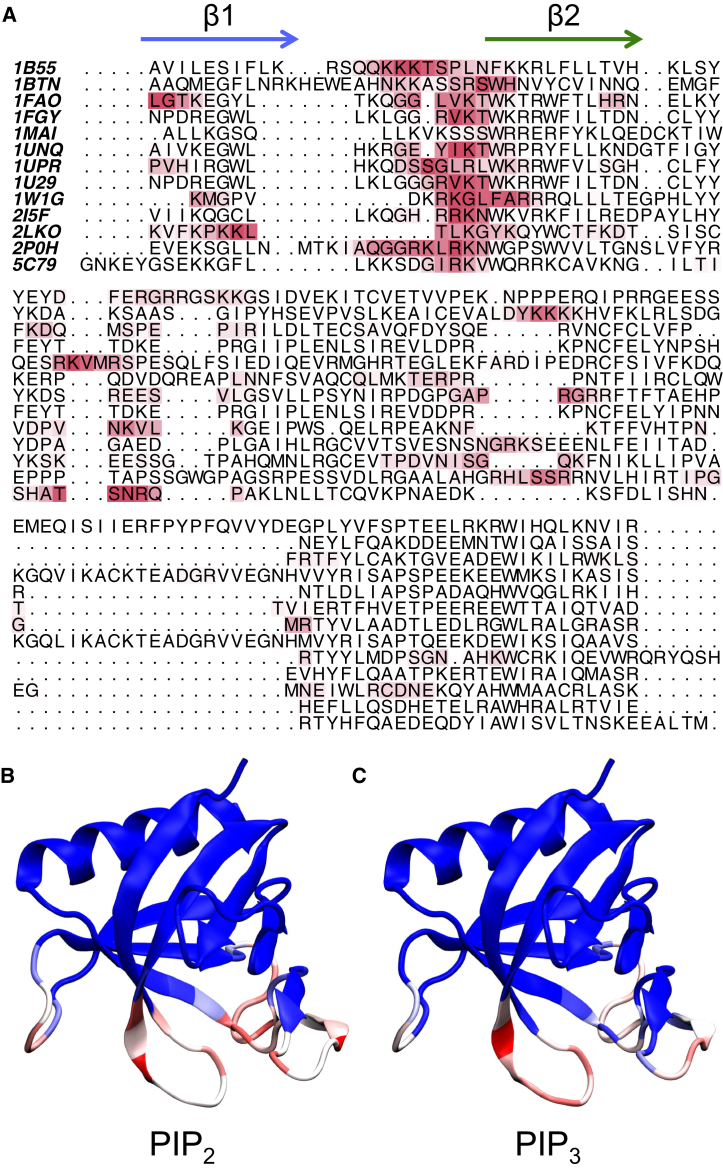
Conservation of the Interactions with PIP Lipids (A–C) Sequence alignment of the PH domains used in this study (A). Red indicates a high number of contacts, whereas white indicate no contacts. Structures of the GRP1 PH domain color-coded based on the number of contact with PIP_2_ (B) or PIP_3_ (C) (both averaged over 25 × 1 μs CG-MD simulations of the GRP1). Blue indicates no contacts, and red a high number of contacts. See also Figure S6.

The contacts to PIP_2_ and PIP_3_ seen in our simulations may be mapped onto a sequence alignment of the PH domains used in our study (Figure 6). Mapping the averaged contacts onto the structure of the GRP1 PH domain confirms that the primary contacts with the PIP lipids occur to the positively charged loop region between strands β1 and β2. This loop region contains many positively charged residues that form the interactions with the PIP lipid headgroups. Considering the structural similarity of all members of the PH domain family of proteins and the fact that we have used PH domains from different proteins, we suggest that the PH/PIP interaction by the β1/β2 loop is a global property of PH domains. For the β-spectrin and ArhGap9 PH domains that bind PIP molecules at non-canonical (Ceccarelli et al., 2007, Moravcevic et al., 2012), the secondary binding region is located in the β5/β6 loop.

## Discussion

In this study, we have shown that a molecular simulation protocol can yield structural data for PH/PIP complexes that are directly comparable to the complexes obtained from NMR and X-ray crystallography. This result is significant from both methodological and biological perspectives. Methodologically, we have shown that a high-throughput coarse-grained simulation approach, generating ensembles of simulations, can be used to study the structural and dynamic features of the association of PMPs with model membranes. This approach provides significant mechanistic details of the formation of the PH/bilayer complexes that are often difficult to obtain using experimental biophysical and structural techniques. Biologically, our results demonstrate that while the PH/PIP interaction occurs primarily via the β1/β2 loop region, in a number of PH domains a secondary (non-canonical) lipid-binding site is seen.

The major finding of our study is that the β1/β2 loop region constitutes the primary PIP-binding site on PH domains. This is in good agreement with experimental data on the GRP1 and ARNO PH domains, which suggest that mutations of the β1/β2 loop residues abolish/reduce the interactions of the aforementioned PH domains with PIP headgroups (Cronin et al., 2004). In particular, abolishment (K273A mutation) and reduction (R277A and K282A mutations) of PIP_3_ binding with GRP1 are observed (Cronin et al., 2004). Mutations on the K173 residue on DAPP1 abolished the binding to 3-phosphoinositides (Dowler et al., 1999). Similarly, mutations of positive residues on the β1/β2 loop of the Bam32 PH domain (Marshall et al., 2000), PKB PH (Thomas et al., 2002), PDK1 PH (Anderson et al., 1998, Sundaresan et al., 2011), Kindlin 2 PH (Liu et al., 2011, Qu et al., 2011), and ArhGap9 PH (Ceccarelli et al., 2007) also resulted in the decrease/abolishment of their interactions with PIP molecules. In our CG-MD simulation with a K173L mutation on the DAPP1 PH domain, we observed a reduction in the interaction with PIP_2_ and an increase in the probability of the PH domain adopting a different orientation on the membrane surface (see Figure S1). This, in combination with the fact that there are many positively charged amino acids (i.e., lysine and arginine) in the loop between β1 and β2 in all PH domains, suggests that this is a general property of lipid-binding PH domains (Carpten et al., 2007, He et al., 2011).

Importantly, our simulation approach is also able to identify secondary (non-canonical) lipid-binding sites on PH domains. In good agreement with a recent crystallographic study of the ASAP1 PH domain, we could identify two PIP-binding sites on the ASAP1 PH domain (Jian et al., 2015), while suggesting a more physiologically relevant orientation of PIP at the non-canonical binding site. Binding of PIP molecules to atypical (or non-canonical) lipid-binding sites has also been suggested for Sim1, Tiam, β-spectrirn, and ArhGap9 PH domains (Anand et al., 2012, Ceccarelli et al., 2007, Hyvönen et al., 1995, Moravcevic et al., 2012). A recent study on 91 yeast PH domains also showed that the presence of PIP lipids is required for the recruitment of PH domains to the membrane. However, the presence of other lipids often regulated their affinity and specificity (Vonkova et al., 2015). This provides evidence that multiple lipid interactions are crucial for the binding of PH domains to membranes. In addition to the PIP binding to secondary binding sites, we also observe a degree of clustering of PIP lipids around the PH domains. This clustering of PIP lipids may reorganize the local lipid environment creating PIP nanodomains. These PIP nanodomains may in turn be important for the clustering/recruitment of other peripheral or integral membrane proteins (van den Bogaart et al., 2011).

The use of a high-throughput methodology ensures effective sampling in the CG-MD simulations. Despite the known approximations of the CG methodology (discussed in, e.g., Marrink and Tieleman, 2013), the current study provides a paradigm for how a computational pipeline may be used to systematically study and quantify the interactions of multiple members of a family of membrane proteins. Our high-throughput approach makes it easy to simulate not only large number of proteins but also to explore effects of changes in the lipid environment. Given the ongoing increase in the available computational resources, this approach is readily scalable to all the structures of the PH domains for which there are structures in the PDB. It can also be easily extended to a wider range of PMPs, e.g., proteins containing C2 or FERM domains. Recently, similar pipelines have been developed for studying the oligomerization of TM helices (Wassenaar et al., 2015), the interaction of PIP lipids with human RTKs TM and juxtamembrane regions (Hedger et al., 2015), and the insertion of integral membrane proteins into bilayers (Stansfeld et al., 2015). This further proves the feasibility of membrane protein simulation pipelines, and demonstrates that they can be extended to other a variety of membrane protein systems.

## Experimental Procedures

### Coarse-Grained Molecular Dynamics Simulations

CG-MD simulations were performed using GROMACS-4.5.5 (Hess et al., 2008) (also see www.gromacs.org) with the Martini 2.1 force field (Marrink et al., 2007, Monticelli et al., 2008). The simulation systems are shown in Figure S1. The bilayer used in the simulations was comprised of 259 palmitoyloleoylphosphatidylcholine (POPC) (73%), 71 palmitoyloleoylglycerophosphoserine (POPS) (20%), 18 PIP_2_ (5%), and 8 PIP_3_ (2%) lipid molecules. Note that PIP_2_ refers to PI(4,5)P_2_ and PIP_3_ refers to PI(3,4,5)P_3_. Each leaflet thus contained 9 PIP_2_ and 4 PIP_3_ molecules. The systems were solvated with ∼14,000 CG water molecules, and NaCl ions at 150 mM concentration were added to neutralize the system. Flexible loop regions missing from the PH structures and a mutation on the DAPP1 PH domain (K173L) were modeled using MODELLER (Fiser and Šali, 2003). All systems were energy minimized for 200 steps, and equilibrated for 1 ns with the protein backbone particle restrained. For each repeat simulation within an ensemble, the protein was rotated around the *x*, *y*, and *z* axes to form a different initial configuration. For each system an ensemble of 25 simulations of 1.0 μs each were run with a time step of 20 fs. An elastic network model was applied to all backbone particles with a cut-off distance of 0.7 nm to model secondary and tertiary structure (Atilgan et al., 2001). The bond length was constrained to equilibrium lengths using the linear constraint solver (LINCS) algorithm (for molecular simulations) (Hess et al., 1997). Lennard-Jones interactions were shifted to zero between 0.9 and 1.2 nm and Coulombic interactions between 0 and 1.2 nm, respectively. The pressure of 1 bar and temperature of 323 K were controlled using the Berendsen algorithm (Berendsen et al., 1984) with a coupling time of 1 ps.

### Atomistic Molecular Dynamics Simulations

Conversion of CG to atomistic systems was made using a fragment-based approach (Stansfeld and Sansom, 2011a). We performed 2 × 1.0 μs MD simulations for each system of the GRP1 and ASAP1 PH domains and 1 μs MD simulations for each of the PLC-δ1 and β-spectrin PH domains. For the initial configurations, we picked up from 1 μs CG-MDs where the PH domain was in preferable orientation and had a bound PIP at the experimentally suggested binding site. The GROMOS96 43a1 force field (Scott et al., 1999) was used with simple point charge water molecules using GROMACS-4.5.5 software. The temperature of 323 K was controlled using the velocity rescaling method (Bussi et al., 2007) with a coupling time of 0.1 ps. The pressure of 1 bar was controlled with semi-isotropic pressure coupling using the Parrinello-Rahman barostat (Parrinello and Rahman, 1981) with a coupling time of 1 ps. Bond lengths were constrained to equilibrium lengths using the LINCS method. The time step was set at 2 fs. The particle mesh Ewald method was used, with a specified direct space cut-off distance of 1.0 nm.

### Density Maps of the Orientation of the PH Domains Relative to the Lipid Membrane

To investigate orientation of the PH domains relative to the lipid membrane, we consider the density map of the PH domains. Here, we have calculated the 2D normalized histogram of *R*_*zz*_ and *d*_*z*_, where *d*_*z*_ is the perpendicular distance between the centers of mass of a PH domain and the lipid membrane, and where *R*_*zz*_ is the *zz* component of the rotational matrix required for least squares fitting of a protein orientation onto a reference orientation. *R*_*zz*_ was calculated using the *g_rotmat* command in GROMACS. The value of *R*_*zz*_ in the density map varies depending on the reference orientation of the PH domain relative to the membrane. The change in the normalized density map of system can be calculated from

Δ*D*(*R*_*zz*_,*d*_*z*_) = *ρ*(*R*_*zz*_,*d*_*z*_)/*ρ*_0_,

where *ρ*(*R*_*zz*_,*d*_*z*_) and *ρ*_0_ are probabilities at a bin (*R*_*zz*_,*d*_*z*_) and a reference point (which corresponds to the global maximum), respectively. Note that prior to the calculation, the rotation and translation of the protein in the *xy* plane was fitted using the *trjconv* command in GROMACS (Hess et al., 2008). The ensemble used for the calculation is 25 × 1.0 μs for CG-MD, 2 × 1.0 μs for AT-MD of GRP1 and ASAP1, and 1 × 1.0 μs for AT-MD of PLC-δ1 and β-spectrin. Similar methodologies were previously used to calculate the energy landscapes of single-tail lipid flip-flop (Arai et al., 2014) and of the insertion of hydrophobic peptides to model membrane (Ulmschneider et al., 2011).

## Author Contributions

E.Y. and A.C.K. performed the calculation and analysis. The research reported here emerged from lively discussions between E.Y., A.C.K., K.Y., and M.S.P.S. All authors contributed to writing the manuscript.

## Figures and Tables

**Table 1:** Summary of Coarse-Grained Simulations

Protein	PDB	S1, Association	S2, Binding Site	S3, Binding Mode
GRP1	1FGY	24	24	24
ARNO	1U29	25	25	24
PEPP1	1UPR	25	23	18
DAPP1	1FAO	25	24	15
DAPP1 (K173L)	1FAO (K173L)	25	15	6
Btk	1B55	25	23	23
PLC-*δ*1	1MAI	24	24	18
PDK1	1W1G	25	23	20
C-PH	2I5F	24	24	17
Kindlin-2	2LKO	24	17	14
PKB/Akt	1UNQ	24	21 C, 13 non-C	15
β-Spectrin	1BTN	25	21 C, 11 non-C	22
ArhGAP9	2P0H	25	20 C, 15 non-C	23
ASAP1	5C79	24	17 C, 17 non-C	17

For each PH domain, 25 × 1 μs simulations were performed. These have been scored at 1 μs as follows: S1, number of simulations in which the PH domain associates with the lipid bilayer; S2, number of simulations in which a PIP molecule binds to the canonical (C) and non-canonical (non-C) sites on the PH domain; and S3, number of simulations in which a PIP molecule binds to either the canonical or the non-canonical site and the PH domain adopts a canonical orientation relative to the membrane. See also Figures S5 and S6.

## Supplemental Information

Document S1. Figures S1–S7

Document S2. Article plus Supplemental Information
