# Supplementary material for: Interactions of Pleckstrin Homology Domains with Membranes: Adding Back the Bilayer via High-Throughput Molecular Dynamics
Source: Structure. 2016 Aug 2;24(8):1421–31. doi: 10.1016/j.str.2016.06.002 (PMC4975593; doi:10.1016/j.str.2016.06.002)
Supplement: Document S1. Figures S1–S7 [file mmc1.pdf]

Structure, Volume *24*

Supplemental Information

Interactions of Pleckstrin Homology Domains with Membranes: Adding Back the Bilayer via High-Throughput Molecular Dynamics

Eiji Yamamoto, Antreas C. Kalli, Kenji Yasuoka, and Mark S.P. Sansom

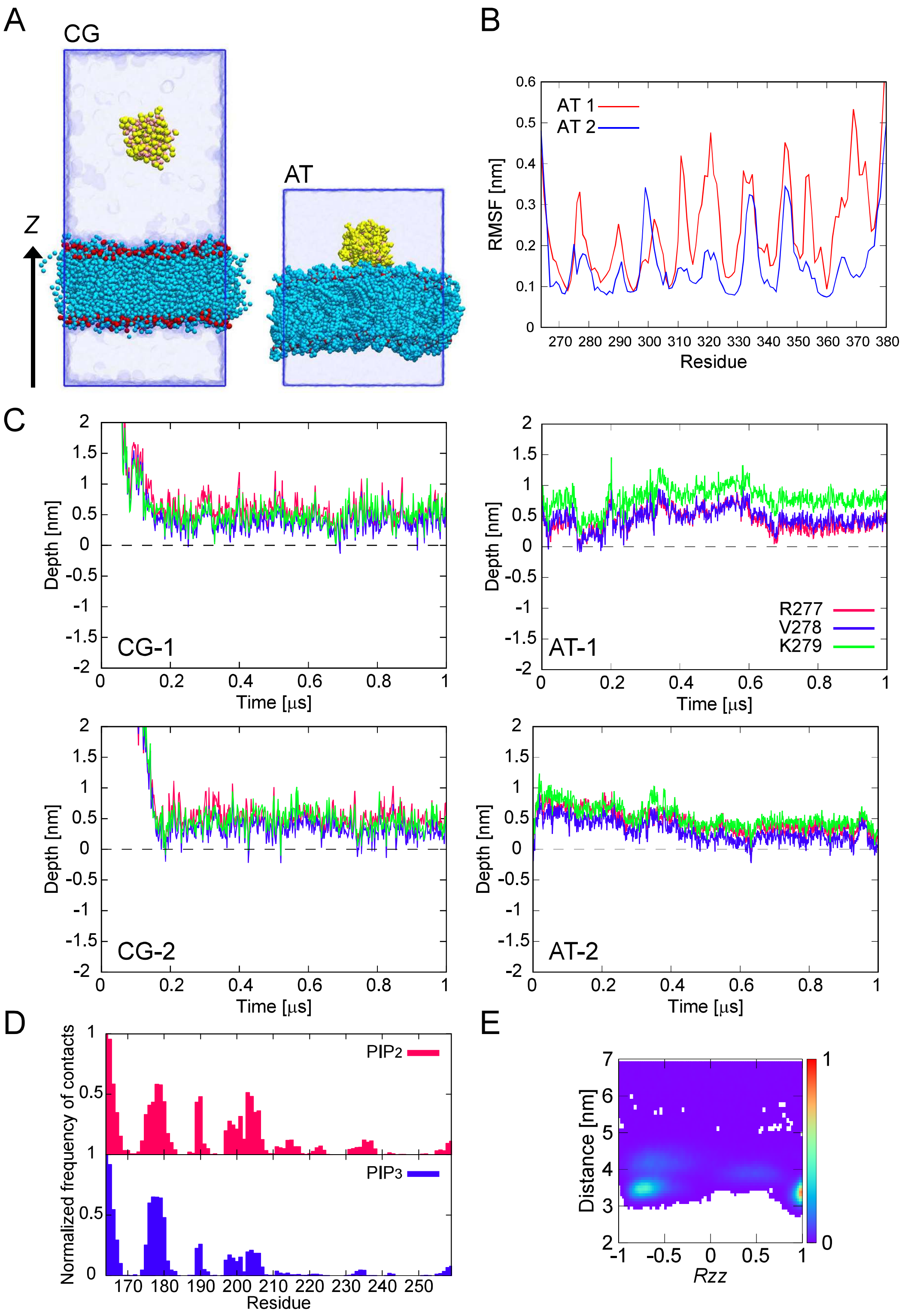

Figure S1, related to Figures 1 and 2. PH domain/bilayer simulations and analysis.
(A) The initial system used for MD simulations. The PH domain and lipid bilayer are shown in yellow (Cα atom: pink) and light blue (phosphate particle: red), respectively. Water molecules are indicated by the upper and lower transparent coatings. The blue line of the box defines the unit cell. The system sizes $L_x \times L_y \times L_z$ are CG: 10.9×10.9×20.1 nm^3 and AT: 10.5×10.5×12.9 nm^3. (B) Root Mean Square Fluctuation (RMSF) analysis for GRP1 PH domain for the AT-MD simulations. The

RMSF values were calculated from the last 0.9 μs of AT-MD trajectories. The first 0.1 μs were discarded as "equilibration" periods. (C) Penetration of GRP1 PH domain into the membrane. The depths of the Cα atoms of R277, V278, and K279 residues with respect to the membrane surface (defined by the average position of phosphates of the lipids, shown as a dashed line at 0) were calculated. (D,E) Mutation in the DAPP1 PH domain (K173L). (D) Normalized average number of contacts between the PH domain and PIP molecules are shown for the 25×1 μs CG-MD simulations. The contact of residues around 164-166 and 176-180 with PIP_2 are changed by this mutation (see Fig. 4 in main text). (E) Normalized density map for the PH domain (*zz* component of rotational matrix *vs* distance). The state around *Rzz* = -0.8 increased by this mutation compared with that of the WT (see Fig. 3 in main text).

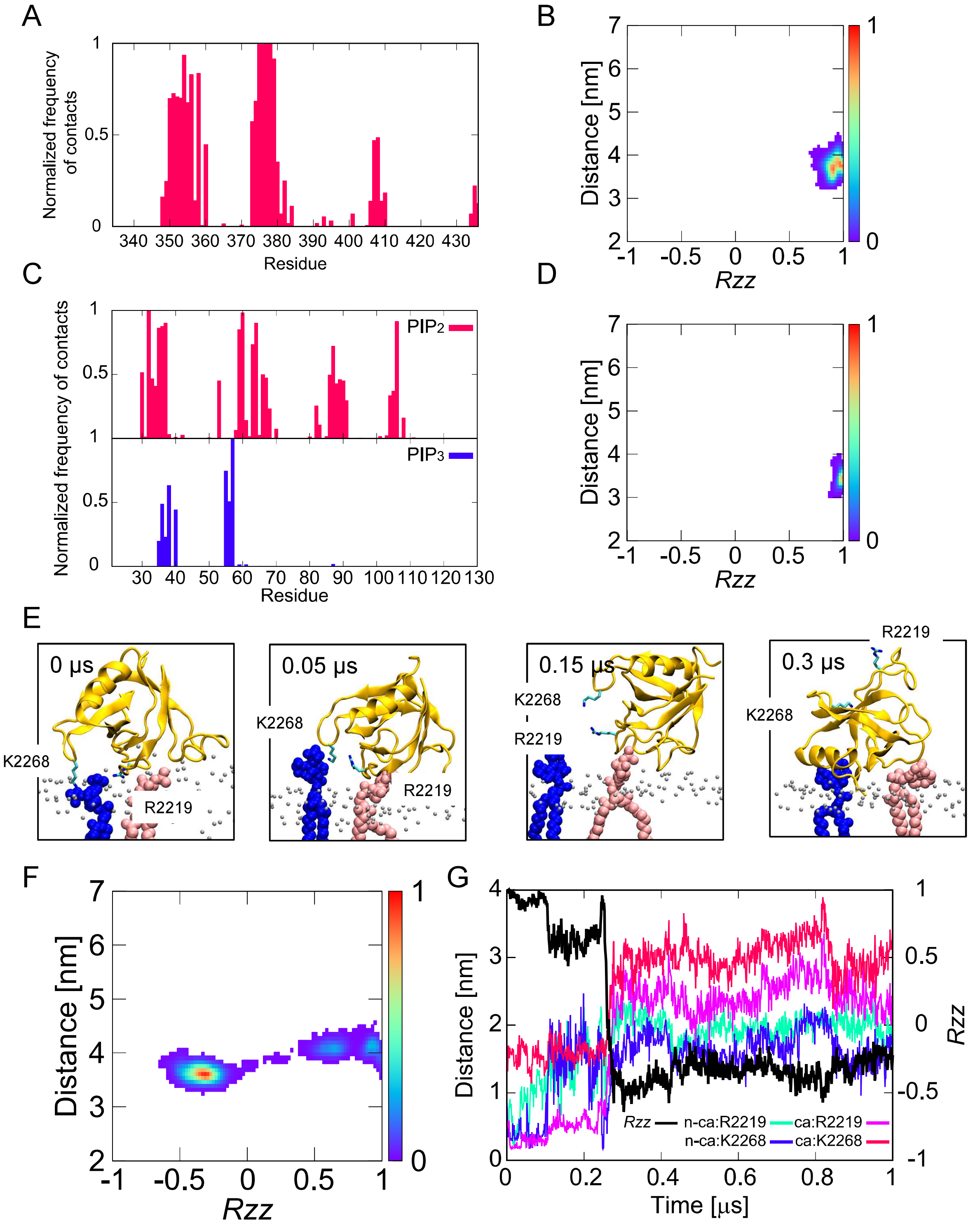

Figure S2, related to Figures 1 and 5. Atomistic simulations of PH domains

(A,B) AT-MD of the ASAP1 PH domain. (A) Normalized average number of contacts between the PH domain and PIP_2 (cutoff distance 0.4 nm) shown for the 2×1 µs AT-MD simulations. (B) Normalized density map for the PH domain (*zz* component of rotational matrix *vs* distance). (C,D) AT-MD of the PLC-δ1 PH domain. (C) Normalized average number of contacts between the PH domain and PIPs (cutoff distance 0.4 nm) are shown for the 1 µs AT-MD simulation. (D) Normalized density map for the PH domain (*zz* component of rotational matrix *vs* distance). (E-G)

AT-MD of the β-spectrin PH domain. (E) Snapshots of one of the atomistic simulations with the PH domain (yellow) with PIPs in the non-canonical (blue) and canonical (pink) sites. The phosphate atoms of lipids are coloured in silver. (F) Normalized density map for the PH domain (*zz* component of rotational matrix *vs* distance) for the 1 μs AT-MD simulation. (G) R_{zz} (black solid line) and minimum distances between PIPs and the amino acid (R2219 and K2268) relative to the simulation time: green and blue lines show PIPs that are in the non-canonical site and pink and red lines show PIPs that are in the canonical site. When the PIP unbinds from the non-canonical site, the orientation of the PH domain changes to a different orientation. Interestingly, this orientation corresponds to a secondary orientation that we observe in the CG-MD simulation with the β-spectrin PH domain (see Fig. 3 in the main text). This suggests that our AT-MD simulation is long enough to see the different membrane-bound states (at the atomistic resolution) that we observe in the CG-MD simulations.

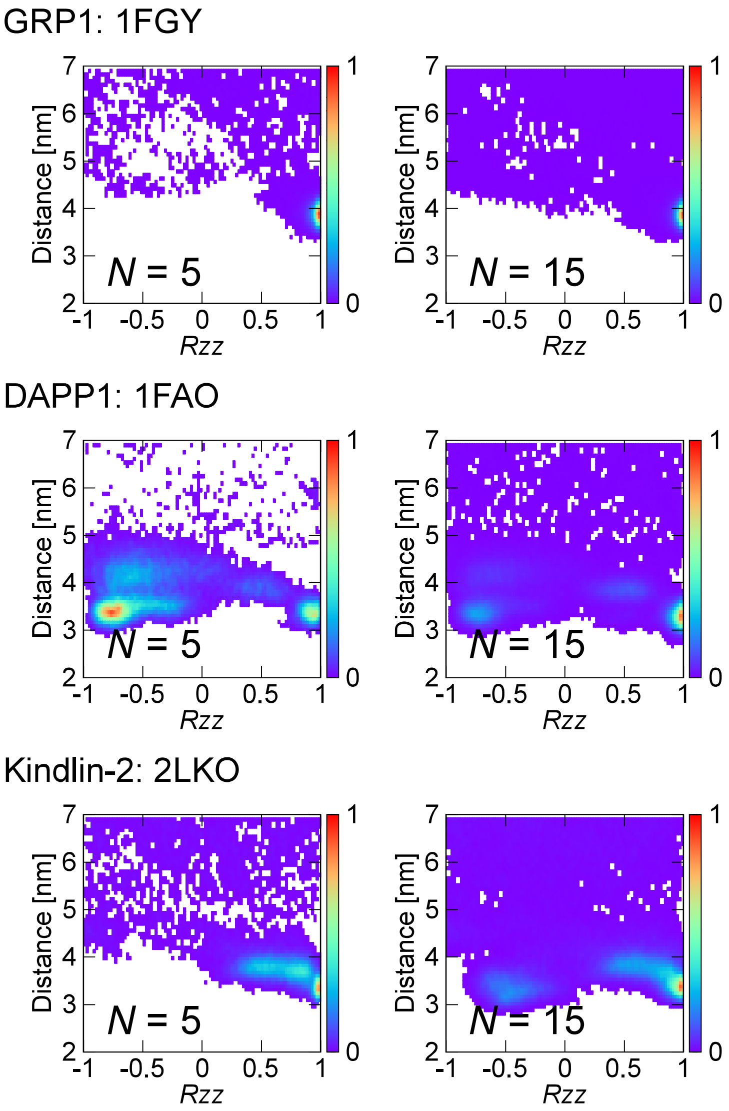

Figure S3, related to Figure 2. Convergence analysis of normalized density maps

The *zz* component of the rotational matrix (*Rzz*) *vs* the distance between centres of mass of the PH domain and the bilayer for the GRP1, DAPP1, and Kindlin-2 PH domains is shown for the ensemble when we have used 5 and 15 repeat simulations to construct the landscape. Those with 25 ensembles are shown in main text (see Fig. 3). This analysis suggests that the density landscape of the 25 ensembles was converged.

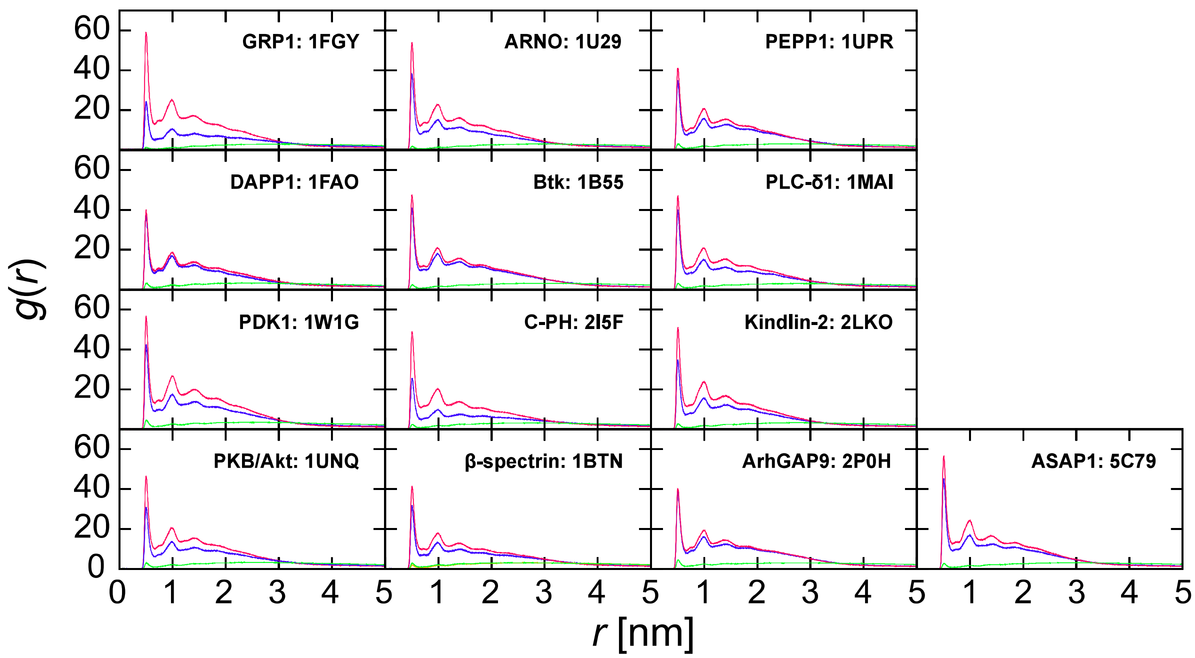

Figure S4, related to Figure 4. Clustering of lipid molecules around the PH domains

Radial distribution function $g(r)$ of lipid molecules around the PH domains derived from the CG-MD simulations. Red, blue, and green coloured lines represent the distribution of PIP_2, PIP_3, and POPS, respectively.

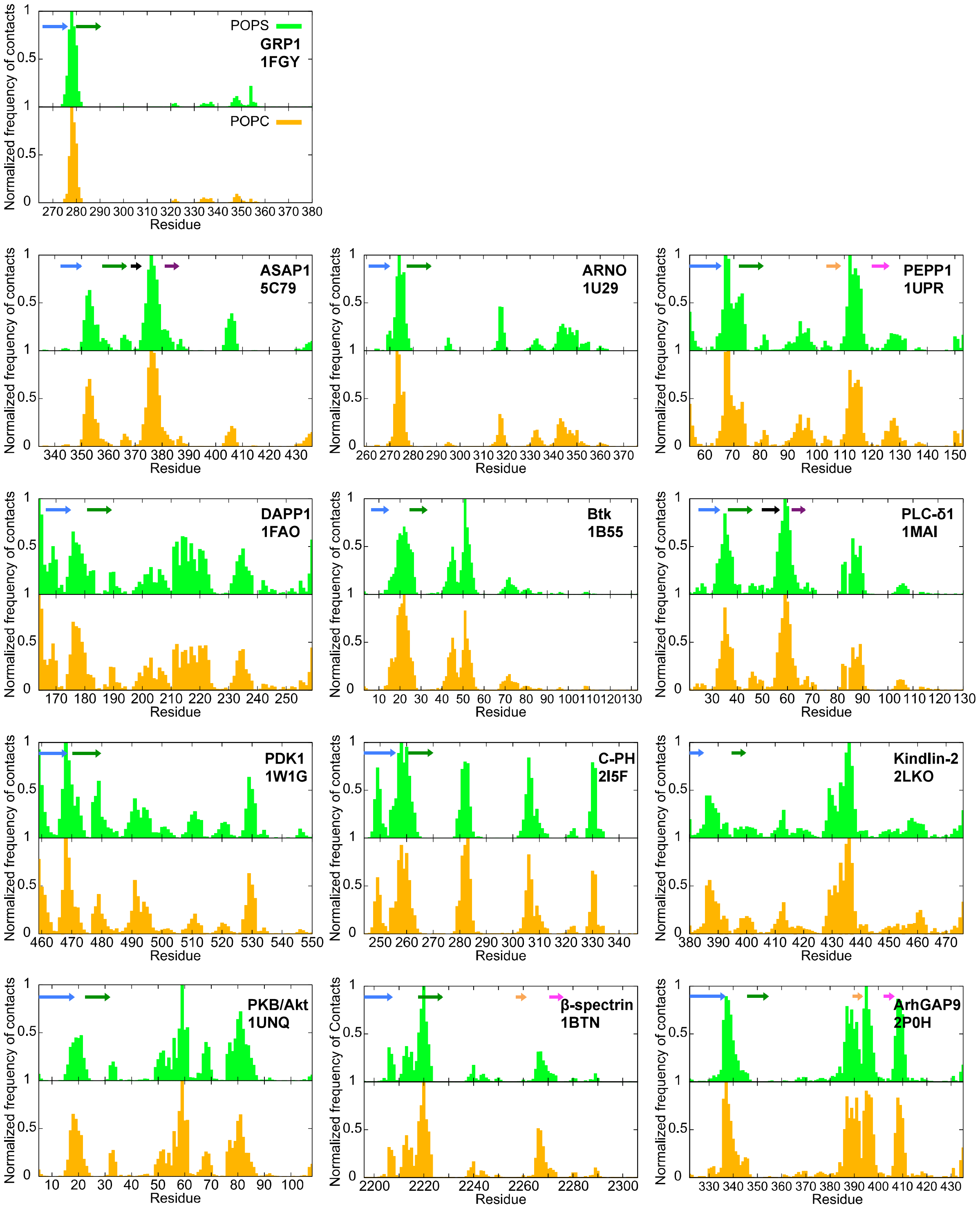

Figure S5, related to Figure 4. Normalized average number of contacts between PH domains and bilayer lipids

Contacts are shown for POPS (green) and POPC (yellow), respectively. The data are averaged over 25×1 μs CG-MD simulations.

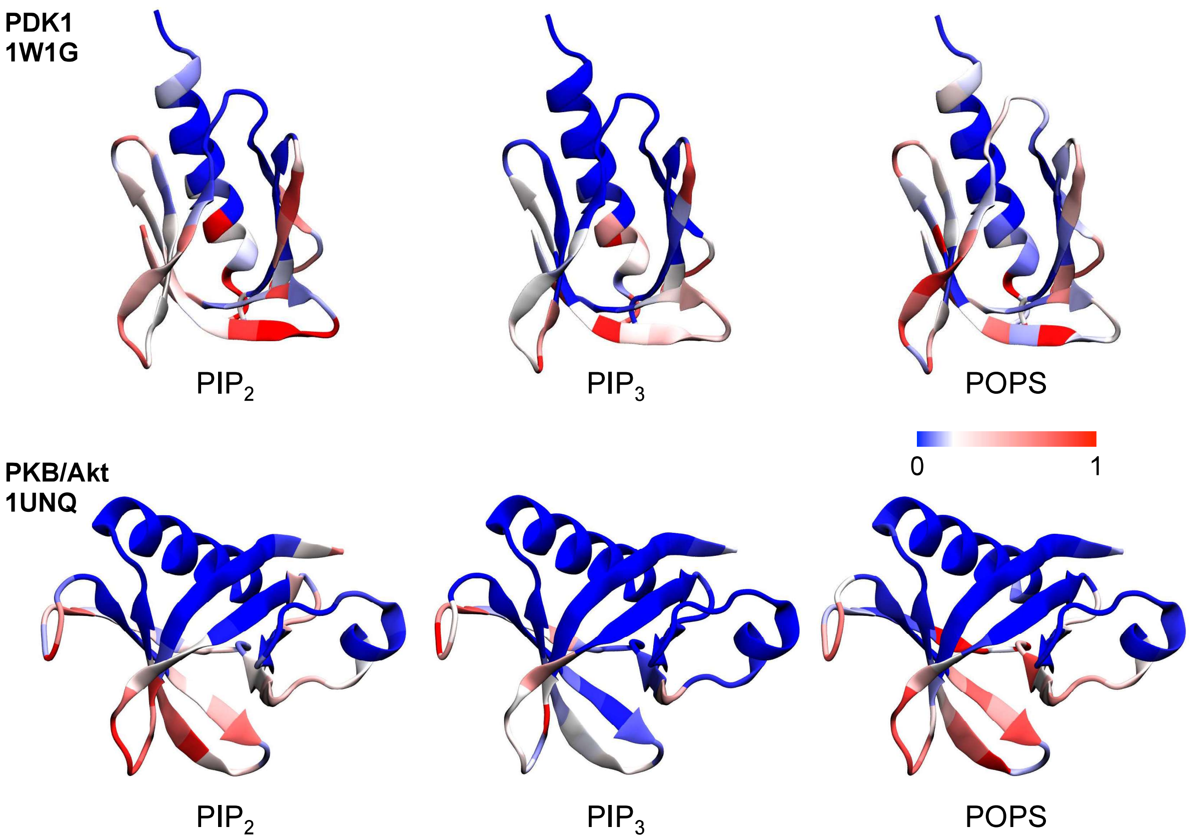

Figure S6, related to Figure 6. Contacts between PH domains and lipids

The amino acids are color-coded based on the normalized average number of contacts with the PIP_2, PIP_3, and POPS. Blue indicates a low number of contacts (or no contacts) and red a high number of contacts.

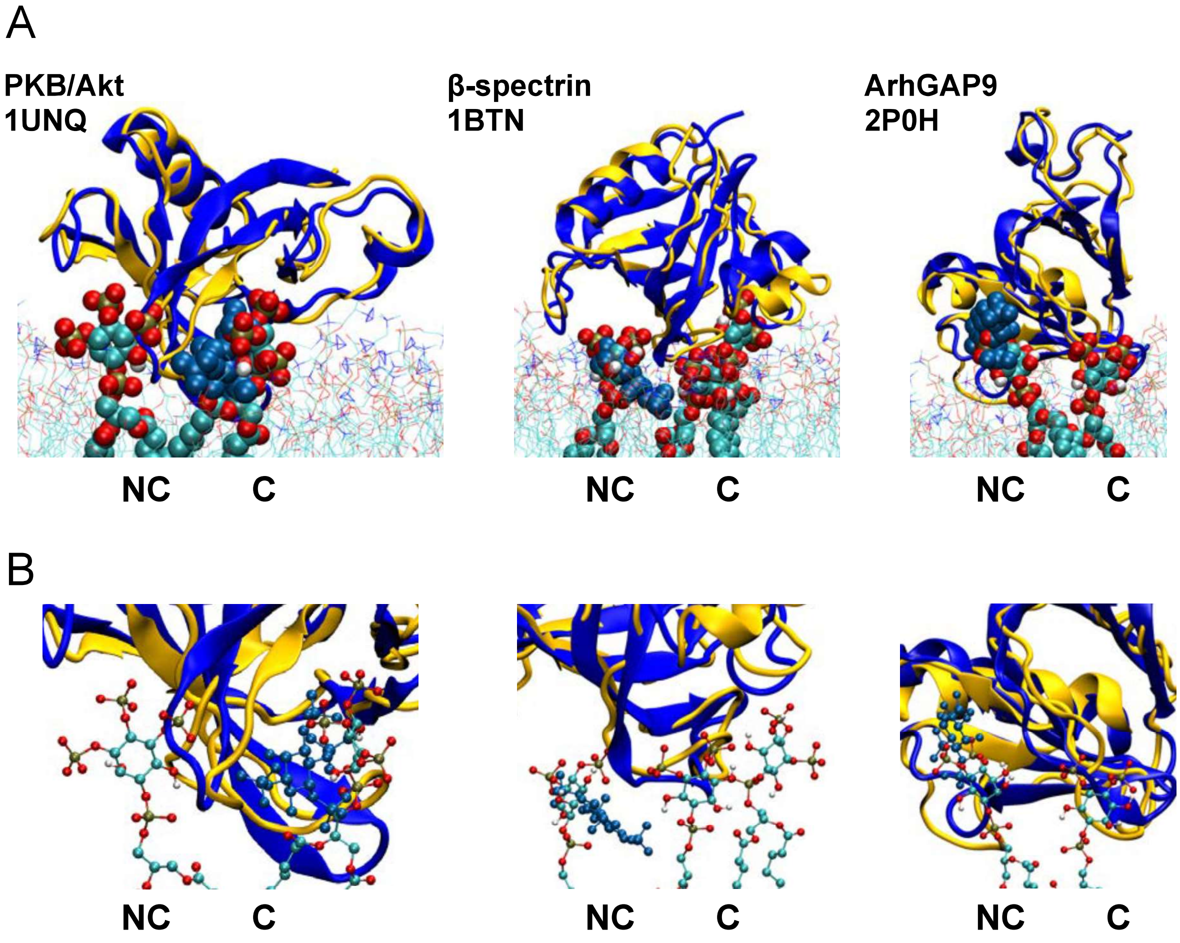

Figure S7, related to Figure 3. PIP lipids at canonical (C) and non-canonical (NC) binding sites

Alignment of the PH/PIP complex derived from our simulation approach (PH domain in yellow and PIP in cyan/red/bronze) with the crystal structure (PH domain and PIP both in blue). The figures in **B** show zoomed in versions of the figures in **A**, focussing on the bound PIP molecules.
