# Supplementary material for: Interactions of Pleckstrin Homology Domains with Membranes: Adding Back the Bilayer via High-Throughput Molecular Dynamics
Source: Structure. 2016 Aug 2;24(8):1421–31. doi: 10.1016/j.str.2016.06.002 (PMC4975593; doi:10.1016/j.str.2016.06.002)
Supplement: Document S2. Article plus Supplemental Information [file mmc2.pdf]

Theory

Structure

Interactions of Pleckstrin Homology Domains with Membranes: Adding Back the Bilayer via High-Throughput Molecular Dynamics

Graphical Abstract

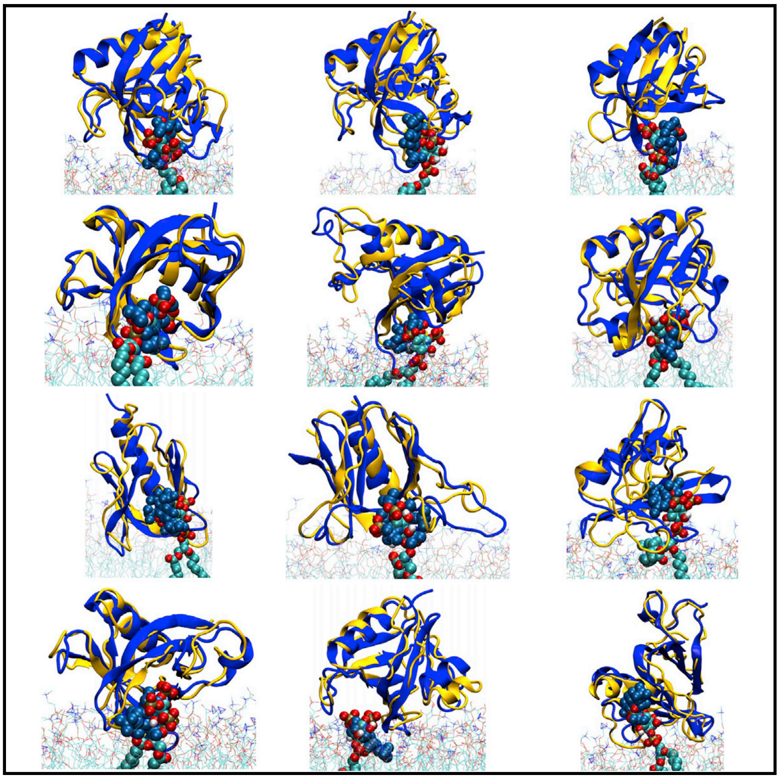

Highlights

- Interactions of 13 different PH domains with PIP-containing membranes are simulated
- PH domains associate with PIPs in membranes via a positively charged loop
- Binding of PH domains causes clustering of PIP molecules within membranes
- Simulations reveal canonical and alternative binding modes of PIPs to PH domains

Authors

Eiji Yamamoto, Antreas C. Kalli, Kenji Yasuoka, Mark S.P. Sansom

Correspondence

antreas.kalli@bioch.ox.ac.uk (A.C.K.),
mark.sansom@bioch.ox.ac.uk (M.S.P.S.)

In Brief

Using a molecular simulation pipeline Yamamoto et al. reveal the interactions of 13 PH domains with PIP-containing lipid bilayers. Some PH domains exhibit modes of interaction with PIP-containing membranes additional to the canonical binding mode. All the PH domains cause a degree of local clustering of PIP molecules upon binding to the membrane.

Yamamoto et al., 2016, Structure 24, 1421–1431
August 2, 2016 © 2016 The Authors. Published by Elsevier Ltd.
http://dx.doi.org/10.1016/j.str.2016.06.002

CellPress

Interactions of Pleckstrin Homology Domains with Membranes: Adding Back the Bilayer via High-Throughput Molecular Dynamics

Eiji Yamamoto,[1] Antreas C. Kalli,[2,*] Kenji Yasuoka,[1] and Mark S.P. Sansom[2,*]
[1]Department of Mechanical Engineering, Keio University, 3-14-1 Hiyoshi, Kohoku-ku, Yokohama-shi, Kanagawa-ken 223-8522, Japan
[2]Department of Biochemistry, University of Oxford, South Parks Road, Oxford OX1 3QU, UK
*Correspondence: antreas.kalli@bioch.ox.ac.uk (A.C.K.), mark.sansom@bioch.ox.ac.uk (M.S.P.S.)
http://dx.doi.org/10.1016/j.str.2016.06.002

SUMMARY

A molecular simulation pipeline for determining the mode of interaction of pleckstrin homology (PH) domains with phosphatidylinositol phosphate (PIP)-containing lipid bilayers is presented. We evaluate our methodology for the GRP1 PH domain via comparison with structural and biophysical data. Coarse-grained simulations yield a 2D density landscape for PH/membrane interactions alongside residue contact profiles. Predictions of the membrane localization and interactions of 13 PH domains reveal canonical, non-canonical, and dual PIP-binding sites on the proteins. Thus, the PH domains associate with the PIP molecules in the membrane via a highly positively charged loop. Some PH domains exhibit modes of interaction with PIP-containing membranes additional to this canonical binding mode. All 13 PH domains cause a degree of local clustering of PIP molecules upon binding to the membrane. This provides a global picture of PH domain interactions with membranes. The high-throughput approach could be extended to other families of peripheral membrane proteins.

INTRODUCTION

The association of peripheral membrane proteins (PMPs) with cell membranes is crucial for many cellular functions, including cell signaling and trafficking (Cho and Stahelin, 2005). This association is often mediated by lipid-binding modules, e.g., the pleckstrin homology (PH) domain found in many PMPs (Lemmon, 2008). Determining the interactions of such domains with the membrane at the molecular level is central to our understanding of the function of PMPs. PMPs interact with the surface of cell membranes via a mixture of specific and non-specific interactions, which sometimes include contributions from covalently attached lipid anchors (Hancock, 2003). Association of PMPs with cell membranes is often controlled by binding to specific lipids, e.g., to phosphatidylinositol phosphates (PIPs) present in cell membranes (Balla, 2005; Stahelin et al., 2014).

The number of PMPs structures has increased significantly during recent years. For example, there are currently ~150 structures of PH domains deposited in the PDB. Although numerous PMP structures have been determined, both by X-ray crystallography and by nuclear magnetic resonance (NMR), only rarely do such structures reveal directly the nature of their interactions with membranes. Indeed, structures of PMPs often do not contain bound lipid molecules. Even when there is a lipid molecule bound in a crystal structure, it is often simply the head group of the lipid that is bound to the PMP. This provides a radically simplified model of the in vivo environment in which PMPs function, and provides only indirect indications as to their exact position and orientation on a cell membrane. Using such structural data, it remains challenging to understand the mechanistic details of their association with membranes and of their interactions with lipid molecules that may be also coupled with conformational changes within the protein and penetration of parts of the protein into the bilayer. We, therefore, need to characterize the modes of interaction of PMPs with their target cell membranes in order to understand the relationship between their molecular structure and biological function. Biophysical studies (e.g., NMR and single-molecule fluorescence microscopy; Knight et al., 2010; Kutateladze and Overduin, 2001; Stahelin et al., 2014) can provide some information, but such detailed characterization is not available for the majority of PMPs. This is especially likely to be the case as higher-throughput experimental approaches are used to explore the interactions between membranes and PMPs (Best, 2014; Vonkova et al., 2015).

Molecular dynamics simulations provide a computational approach to characterize the interactions of membrane proteins with their lipid bilayer environment (Stansfeld and Sansom, 2011b), and in particular to study the interactions of PMPs with model membranes (Kalli and Sansom, 2014; Vermaas et al., 2015). Recently, high-throughput molecular dynamics simulations have been used to study, e.g., the oligomerization of TM helices (Wassenaar et al., 2015), the association of phosphatase and tensin homolog with model membranes (Kalli et al., 2014), or anomalous dynamics of DAPP1 PH domain on model membranes (Yamamoto et al., 2015). Comparisons with experiments have shown that these simulations are in good agreement with available experimental data. In this study we present a high-throughput molecular dynamics simulation protocol that allows us to study the interaction of PMPs with model membranes. This approach was applied to a family of PMPs for which we have many structural and functional data, i.e., the PH domains.

This is an open access article under the CC BY license (http://creativecommons.org/licenses/by/4.0/).

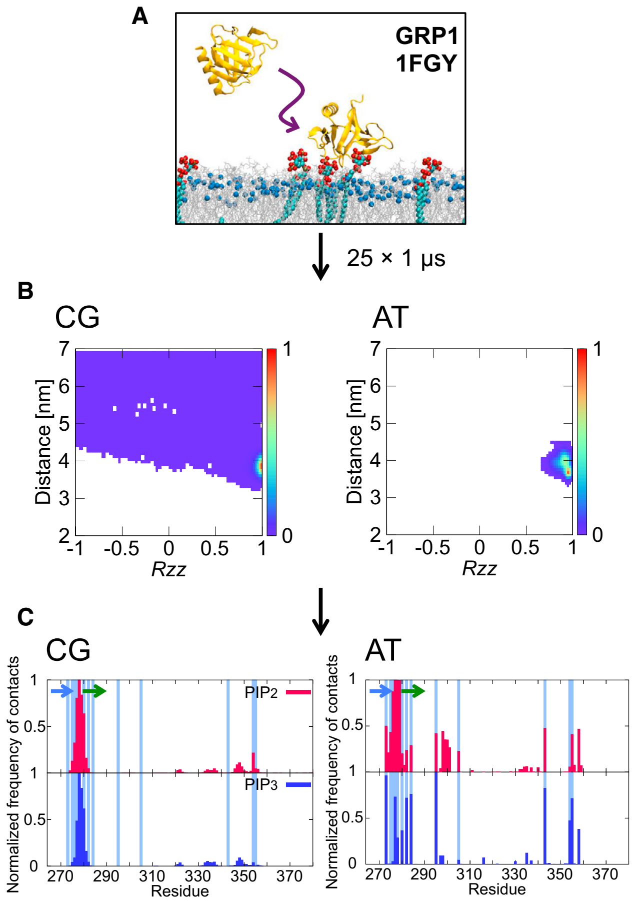

Figure 1. The PH Domain/Bilayer Simulation Pipeline

(A) Snapshot of a selected simulation demonstrating the localization of the GRP1 PH domain to the lipid bilayer. The GRP1 PH domain is shown in yellow. PIP_3 molecules are shown in green/red/bronze, and the POPC and POPS lipids are shown as silver lines (phosphorus atoms, blue). See also Figure S1.

(B) Normalized density map of the GRP1 PH domain (*zz* component of rotational matrix versus distance).

(C) Normalized average number of contacts between the GRP1 PH domain protein and PIPs shown for the 25 × 1 μs CG-MD simulations and for the 2 × 1 μs AT-MD simulations (see also Figure S1). The light blue colors represent the experimental contacts observed in the crystal structure. For normalization, the number of contacts of a residue with a lipid type was divided by the largest number of contacts that the same lipid type made with any residue in the protein. This means that the residue with the most frequent contacts will have the value of 1 and the residue with no contacts with a lipid type will have the value of 0. The position of the β1 and β2 strands is shown by blue and green arrows, respectively. Contacts were defined using cut-off distances of 0.7 and 0.4 nm, respectively for CG-MD and AT-MD simulations. The same analysis for the atomistic simulations of the PLC-δ1 PH and the β-spectrin PH domains is shown in Figure S2.

PH domains are an important class of membrane recognition domains that bind to specific lipids (PIPs) in cell membranes. Many structures of PH domains are known, some (ca. 13) with bound inositol-phosphates (IPs, i.e., PIP headgroups). Each PH domain consists of ~120 residues with an antiparallel β sheet architecture followed by one or two amphipathic α helices (Stahelin et al., 2014). The majority of PH domains have a KXn(K/R)XR motif in the loop connecting strands β1 and β2. This positively charged sequence was shown to regulate the contacts of the PH domains with different types of PIP molecules (Ferguson et al., 2000; Moravcevic et al., 2012). There are, however, a number of PH domains that do not have this consensus sequence, e.g., the β-spectrin and ArhGap9 PH domains (Ceccarelli et al., 2007; Hyvönen et al., 1995; Moravcevic et al., 2012). For these PH domains it was shown that the binding of PIP lipids occurs on the opposite face of the β1/β2 strands. Interestingly, recent studies (Jian et al., 2015; Vonkova et al., 2015) suggest that the nature of the interactions of PH domains with the target membrane may be more complex than simple recognition of a single lipid (PIP) headgroup. A recent structure of the ASAP1 PH domain suggested that PIP may bind to both a canonical site (similar to that found in those PH domains that have the KXn(K/R)XR motif) and to a non-canonical site (similar to the PH domains that do not have the KXn(K/R)XR motif) (Jian et al., 2015). In the PDB there are structures for all three types of PH domains, and thus in our study we have examined the binding to model membranes of all three different types of PH domains. In particular, we have studied GRP1, ARNO, PLC-δ1, DAPP1, PDK1, PEPP1, PKB/Akt, C-PH, Kindlin-2, and Btk PH domains (Baraldi et al., 1999; Cronin et al., 2004; Ferguson et al., 1995, 2000; Jackson et al., 2007; Komander et al., 2004; Lietzke et al., 2000; Liu et al., 2011; Milburn et al., 2003) that do have the KXn(K/R)XR motif (canonical PIP-binding site); ArhGAP9 and β-spectrin PH domains (Ceccarelli et al., 2007; Hyvönen et al., 1995) that do not have the KXn(K/R)XR motif (non-canonical PIP-binding site), and the ASAP1 PH (Jian et al., 2015) domain that is proposed to have both canonical and non-canonical PIP-binding sites.

Here, we present a computational pipeline for studying the interactions of PH domains with PIP-containing membranes. We evaluate this method for the canonical PH domain of GRP1. We derive a 2D density landscape for the protein/membrane interaction alongside residue contact profiles that fingerprint the protein/PIP interactions. We investigate the localization on the surface of a model membrane of 13 different PH domains for which there are structures for the PH/(P)IP complex in the PDB. Our results demonstrate that some PH domains are predicted to have modes of interaction with PIP-containing membranes additional to the canonical binding mode. These studies provide a global picture of PH domain interactions with membranes, and exemplify high-throughput molecular dynamics simulations as a more general protocol for exploring PMP/membrane interactions.

RESULTS

GRP1 PH Domain: a Canonical PH Domain to Develop and Evaluate the Method

Our simulation pipeline (Figure 1) was first tested using the GRP1 PH domain (see Table 1) for which combined biophysical data and atomistic molecular dynamics (AT-MD) simulations have demonstrated the preferred (i.e., canonical) mode of interaction of the PH domain with a PIP_3 molecule in a phospholipid bilayer (Lumb et al., 2011). At the start of each coarse-grained molecular dynamics (CG-MD) simulation, the PH domain was placed in a simulation box at a distance of 7 nm away from a preformed PC/PS/PIP_2/PIP_3 (73%/20%/5%/2%) lipid bilayer. An ensemble

Table 1. Summary of Coarse-Grained Simulations

Protein	PDB	S1, Association	S2, Binding Site	S3, Binding Mode
GRP1	1FGY	24	24	24
ARNO	1U29	25	25	24
PEPP1	1UPR	25	23	18
DAPP1	1FAO	25	24	15
DAPP1 (K173L)	1FAO (K173L)	25	15	6
Btk	1B55	25	23	23
PLC-δ1	1MAI	24	24	18
PDK1	1W1G	25	23	20
C-PH	2I5F	24	24	17
Kindlin-2	2LKO	24	17	14
PKB/Akt	1UNQ	24	21 C, 13 non-C	15
β-Spectrin	1BTN	25	21 C, 11 non-C	22
ArhGAP9	2P0H	25	20 C, 15 non-C	23
ASAP1	5C79	24	17 C, 17 non-C	17

For each PH domain, 25 × 1 μs simulations were performed. These have been scored at 1 μs as follows: S1, number of simulations in which the PH domain associates with the lipid bilayer; S2, number of simulations in which a PIP molecule binds to the canonical (C) and non-canonical (non-C) sites on the PH domain; and S3, number of simulations in which a PIP molecule binds to either the canonical or the non-canonical site and the PH domain adopts a canonical orientation relative to the membrane. See also Figures S5 and S6.

of 25 repeat simulations was performed, with each simulation of duration 1 μs and starting from different initial orientations of the PH domain relative to the bilayer. During the simulations, the PH domain diffuses in the aqueous environment before encountering and forming a complex with the PIP-containing lipid bilayer (Figure 1A).

To quantify the binding of the GRP1 PH domain to the membrane, the progress of each simulation was tracked in terms of the distance from and orientation of the PH domain relative to the lipid bilayer. Merging these data across the whole ensemble allows one to construct a 2D density map describing the interaction of a PH domain with a model membrane (see Experimental Procedures for details). The resultant density map may be described in terms of the number and depth of the density minima. For the GRP1 PH domain there is a single maximum, corresponding to a single preferred orientation of the protein relative to the membrane. This orientation corresponds well with that previously determined by Lai et al. (2013) using atomistic simulations, and is similar to the GRP1/membrane complex derived by Lumb et al. (2011) combining NMR observations and atomistic simulations. We have confirmed the convergence of the density map calculations using different numbers of repeat simulations in the ensemble for three different PH domain systems (see Figure S3). By way of comparison, we note that experimental measurements of the dissociation constant (K_d) of GRP1 PH with PtdIns(3,4,5)P_3 or Ins(3,4,5)P_3 molecules in solution yielded values of 50 nM (corresponding to ca. −10 kcal/mol) (Klarlund et al., 2000) and ~30 nM (Kavran et al., 1998), respectively. A K_d of 50 nM for the GRP1 PH domain binding to PIP_3 in an anionic bilayer (Corbin et al., 2004) was estimated using a fluorescence resonance energy transfer competitive binding assay in which IP_6 molecules were used to dissociate GRP1 PH from bilayers containing PIP_3 molecules and other anionic lipids (i.e., phosphatidic acid).

We have examined the main contacts of the PIP molecules with the bound PH domain, averaged over the ensemble of simulations. We have analyzed these both for the ensemble of CG simulations, and also for the atomistic simulations launched from the preferred CG orientation. Protein/lipid contacts for the GRP1 PH domain with PIP_3 and with PIP_2 are shown in Figure 1C. The main contacts are, as anticipated, with the β1/β2 loop. Comparison of our results with the crystallographically observed contacts for bound Ins(1,3,4,5)P_4 shows good agreement. The β6/β7 loop region also makes some contacts with the PIP molecules. Interestingly, in an NMR study (Lumb et al., 2011) of a PIP_3-bound GRP1 PH domain in dodecylphosphocholine micelles, the protein amide resonances changed not only for the β1/β2 loop residues (around residue 280) but also for residues on the β6/β7 loop (around residue 350), both of which loops observed in the PIP_3 contact analysis from the simulation ensemble. This is also consistent with the contacts seen in atomistic simulations of GRP1 modeled as bound to the head group of PIP_3 in a lipid bilayer (Lumb et al., 2011). The main residues that interact with the PIP_2 and PIP_3 molecules are residues 273, 277, 278, 279, and 343. A recent study by Lai et al. (2013). also suggests that the interactions of the β1/β2 loop, and in particular residues R277 and K279, are important for the PH/PS lipid interactions. However, in our study we did not observe significant penetration of GRP1 V278 into the membrane, i.e., below the plane of phosphate in lipids (see Figure S1). In addition, the PIP-binding site was not flexible in our AT-MD simulations in good agreement with previous simulations of GRP1 bound to PIP_3 (Lai et al., 2013) (see Figure S1). A similar orientation of the GRP1 PH domain relative to the bilayer was also observed in an electron paramagnetic resonance study (Chen et al., 2012). We are therefore confident that the results of the CG-MD protocol for the GRP1 PH/PIP interactions agree well with both experimental measurements and more detailed simulations by AT-MD. However, approximations implicit in the CG-MD simulations make it rather more difficult to analyze in detail the specificity of the PH domains for different PIP species. During the extended AT-MD simulations, the PIP lipid interactions with the PH domains were generally retained, with the exception of the PH domain of β-spectrin. This suggests that currently the AT-MD simulations remain too short to direct analysis of the specificity for different species of PIPs. We note that calculations of mean force potentials may allow us to study the specificity of interactions of PIP molecules with PH domains (Naughton et al., 2016), although this approach is currently only feasible for CG simulation models.

A Comparison of 13 PH Domains

PH domains are a structurally conserved family of proteins, although there is a significant degree of sequence variation within the family. Using our high-throughput pipeline, we have extended our studies to multiple members of the PH family of proteins. Thus, we have selected 13 PH domains from the PDB databank for which there are experimentally derived PH/PIP headgroup structures (Baraldi et al., 1999; Ceccarelli et al., 2007; Cronin et al., 2004; Ferguson et al., 1995, 2000; Hyvönen

et al., 1995; Jackson et al., 2007; Jian et al., 2015; Komander et al., 2004; Lietzke et al., 2000; Liu et al., 2011; Milburn et al., 2003) (i.e., for 12 complexes plus GRP1; see Table 1). Note that in this set of PH domains there are ten PH domains with canonical PIP-binding sites, two PH domains with non-canonical PIP sites, and one that exhibits two binding sites. Simulations were set up for all PH domains in the same manner as for GRP1 in order to study their interactions with and orientation relative to a model PIP-containing lipid bilayer.

Calculation of the density landscapes suggests that all of the PH domains adopt a preferred orientation (i.e., a global maximum) relative to the membrane, such that in this preferred orientation each PH domains has a bound PIP lipid at the binding sites suggested by the crystal/NMR structures of the PH/InsP complexes (Figure 2). Indeed comparison of the preferred orientation from each simulation (i.e., the PH/bilayer complex corresponding to the global maximum in the density landscape) with the experimental structures demonstrated that the PH/PIP complexes derived from our study are very similar to the complexes deposited in the PDB (Figure 3). Interestingly, in 83% of the final membrane complexes obtained by all our simulations (i.e., with 13 PH domains), a PIP molecule binds to the same binding site suggested by the PH/InsP complexes obtained by NMR or X-ray crystallography (see Table 1). We note that in the case of PLC-δ1 we also observe strong interaction of the PIP lipid(s) with the β3/β4 loop that is located next to the canonical β1/β2 loop. Strikingly, for β-spectrirn and ArhGAP9 domains, we observe the binding of the PIP lipid molecule on the opposite side of the β1/β2 loop (i.e., at a non-canonical PH site) as expected from the structural data. This is due to the fact that these PH domains lack a KXn(K/R)XR motif which is found in other PH domains (Ceccarelli et al., 2007; Moravcevic et al., 2012).

Closer examination reveals that some PH domains have more complicated density landscapes than others, despite the fact that for all of them the global maximum corresponds to a PH/bilayer complex in the canonical (i.e., preferred) orientation suggested by the experimental structures. For approximately 80% of the time (averaged across all simulation systems), the protein adopted a preferred orientation relative to the bilayer. In the remainder of the simulations, the PH domain adopted a perturbed orientation relative to the bilayer, but, in some of these simulations, a PIP molecule was still able to bind to the proposed PIP-binding site (see Table 1 for more details). In the simulations of GRP1, ARNO, Btk, PDK1, β-spectrin, and ArhGap9 PH domains, the PH/PIP complex was formed in the preferred orientation relative to the bilayer for more than 80% of the final complexes. In particular, GRP1 and ARNO exhibited only the canonical binding mode. The other PH domains had secondary binding modes. In these secondary binding modes, either the orientation of the PH domain changed slightly while retaining a PIP molecule at the canonical binding site or the PH domain interacted with the bilayer via a different positively charged region on the protein. In particular, in the simulations of the PEPP1, DAPP1, PLC-δ1, C-PH, Kindlin-2, PKB/Akt, and ASAP1, more than 60% of the final complexes were in the preferred orientation and had a bound PIP at the experimentally suggested binding sites. For the rest of these simulations, these PH domains were able to adopt alternative orientations relative to the membrane. However, as discussed above, in some of these cases, the PIP molecule was able to bind to the proposed PIP-binding site. Although such secondary binding modes of PH domains have not been previously discussed in detail, these modes may correspond to more transient modes of interactions before the PH domain adopts a canonical orientation. Consequently, the secondary modes may help to mediate the initial encounter between the PH domain and the membrane. However, we note that we used isolated PH domains in our simulations. The secondary binding modes may be sensitive to the presence or absence of the other domains given that PH domains are usually part of larger multi-domain structures.

Analysis of the contacts between the PH domains and the PIP_2 or PIP_3 molecules suggests that all PH domains associate with the membrane via the positively charged loop connecting the β1 and β2 strands, in either a canonical or a non-canonical fashion (see Discussion above; Figure 4). Interestingly, the PH domains for which we have observed secondary binding modes, e.g., the Kindlin-2 PH domain exhibits additional contacts with other positively charged regions of the proteins. Calculation of the radial distribution functions for all the PH domains suggests that there is also a degree of clustering around the PH domain for both PIP_2 and PIP_3 lipids (see Figure S4). Clustering of PIP molecules has also been observed experimentally (Picas et al., 2014). Using CG-MD simulations, the fluctuation of the cluster size of PIPs around a PH domain was examined and found to exhibit 1/*f* noise (Yamamoto et al., 2015). This clustering also contributes to the additional PIP/protein contacts. In vivo studies of PDK1 (Lucas and Cho, 2011) and PKB/Akt (Huang et al., 2011) PH domains suggest that the interactions of the β1/β2 loop are important for PH/PS lipid interactions. These suggested interactions are also observed in our simulations (see Figures S5 and S6).

Non-Canonical PIP Interactions as Exemplified by the PH Domain of ASAP1

Recently, a crystal structure of the ASAP1 PH domain (PDB: 5C79) was determined in which the authors identified an "atypical" (A) binding site in addition to the "canonical" (C) PIP-binding site (Jian et al., 2015). The presence of an additional site on a PH domain may have regulatory and functional roles. Interestingly, analysis of our simulation with the ASAP1 PH domain also revealed that PIP lipids interacted with both the canonical and the atypical sites suggested by the crystal structure (Jian et al., 2015) (see Figure 5). A detailed atomistic simulation of the ASAP1 PH domain confirmed that both binding sites predicted by CG-MD simulations provided stable PH/PIP interactions (see Figure S2). However, inspection of the ASAP1 PH structure suggests that in the crystal the dibutyryl PIP_2 molecule may have adopted an upside down orientation at the A site, as the (short C_4) tails would point away from a bilayer, whereas in our simulations the PIP_2 molecules at both the A and C sites have their alkyl tails pointing toward the membrane.

Binding of PIP molecules to atypical (i.e., non-canonical) lipid-binding sites has also been suggested for Sim1, Tiam, β-spectrirn, and ArhGap9 PH domains (Anand et al., 2012; Ceccarelli et al., 2007; Hyvönen et al., 1995; Moravcevic et al., 2012). These PH domains have the potential for cooperative binding of PIP molecules to canonical and non-canonical sites. There is also an in vivo study that suggests the existence of two lipid-binding

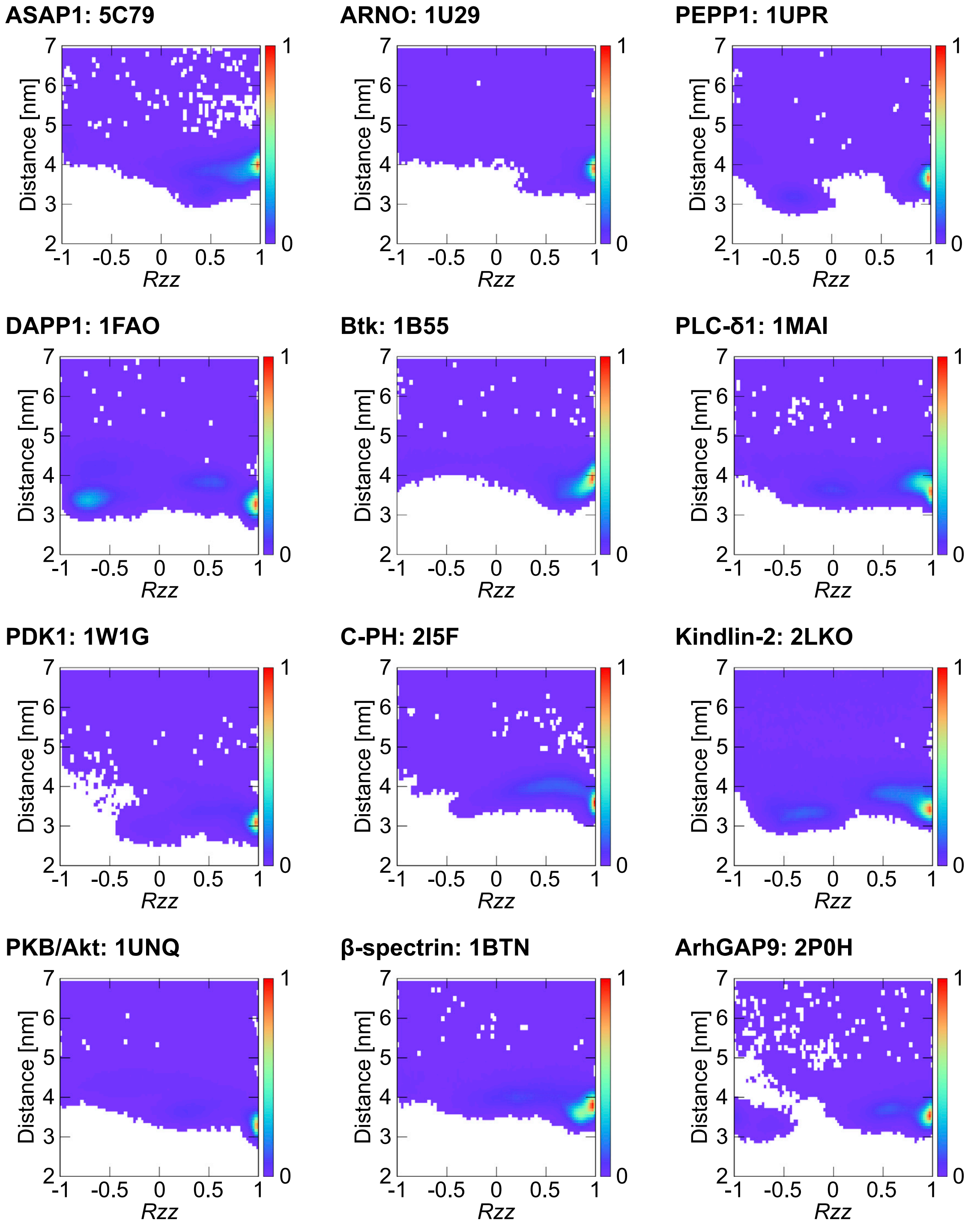

Figure 2. Normalized Density Maps for the 12 PH Domains, Other Than that of GRP1

For the density map of GRP1, see Figure 1C. The normalized density maps are shown as the *zz* component of the rotational matrix versus the *z* component of the distance between centers of mass of the protein and the bilayer. See also Figure S3 for convergence analysis and Figure S1 for the analysis of the orientation of the mutated form of the DAPP1 PH domain relative to the bilayer.

sites of PKB/Akt PH domain (Huang et al., 2011). Overall, we observed binding of PIP molecules to both canonical and non-canonical sites for the PKB/Akt, β-spectrirn, and ArhGap9 PH domains (see Figure S7). Interestingly, in our AT-MD simulation of the β-spectrin PH domain, we observed dissociation of PIP_2 from the non-canonical binding site (see Figure S2). After dissociation, the orientation of the PH domain switched to a different state, corresponding to the secondary orientation seen in the

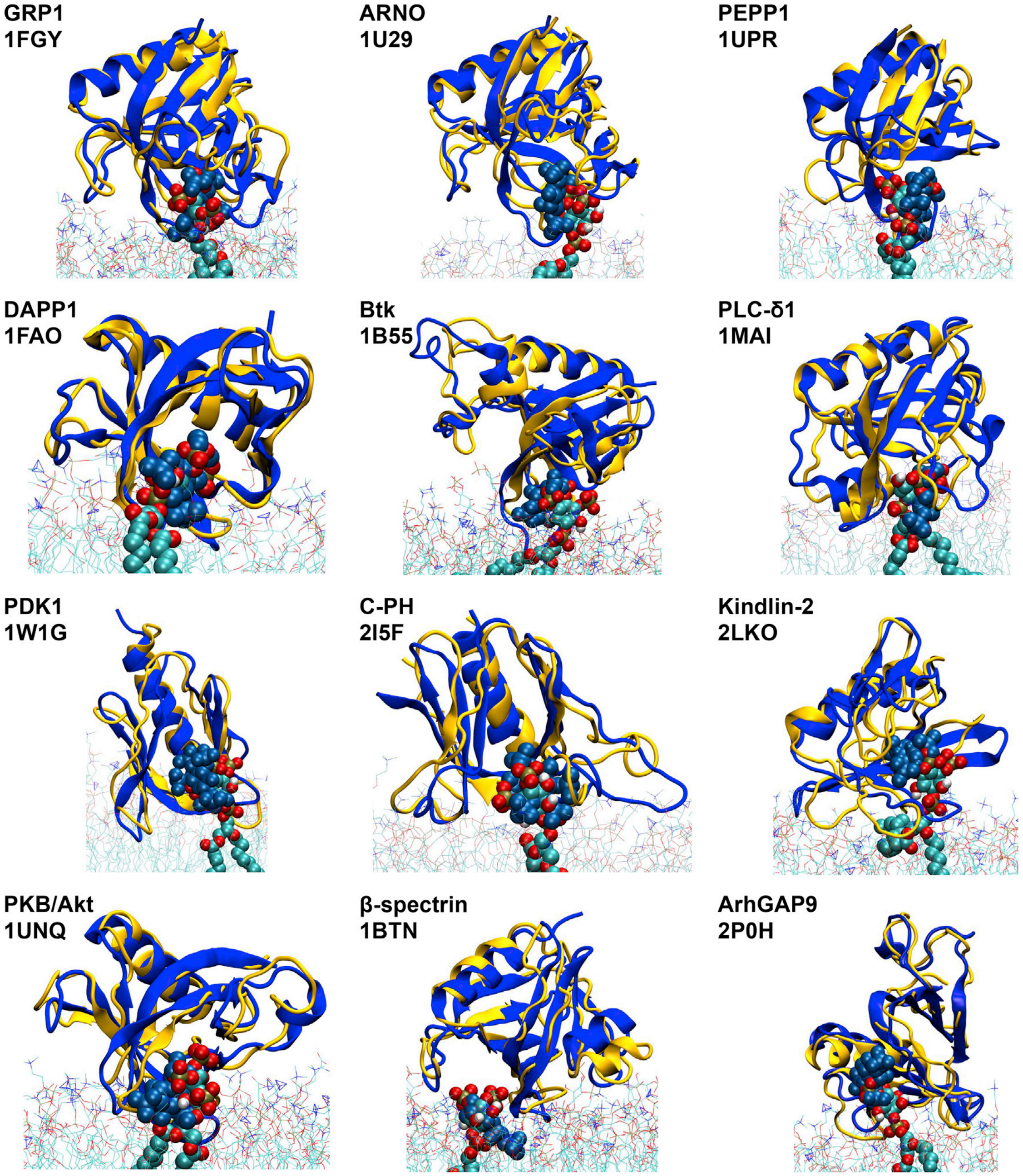

Figure 3. PH/PIP Complexes

Alignment of the PH/PIP complexes derived from our simulation approach (with PH domains in yellow and PIP molecules in cyan/red/bronze/silver) with the corresponding crystal structures (PH domains and PIP both in blue). Note that PIPs in the simulation snapshots are located at approximately the same sites on the PH domains as in the crystal structures. These complexes were obtained from the maxima in the density maps shown in Figure 2. See also Figure S7.

CG-MD simulations. This suggests that the binding via the non-canonical site is important for maintaining the preferable orientation of the PH domain on the membrane surface.

Conservation of the Interactions with PIP Lipids

The contacts to PIP_2 and PIP_3 seen in our simulations may be mapped onto a sequence alignment of the PH domains used in our study (Figure 6). Mapping the averaged contacts onto the structure of the GRP1 PH domain confirms that the primary contacts with the PIP lipids occur to the positively charged loop region between strands β1 and β2. This loop region contains many positively charged residues that form the interactions with the PIP lipid headgroups. Considering the structural similarity of all members of the PH domain family of proteins and the fact that we have used PH domains from different proteins, we suggest that the PH/PIP interaction by the β1/β2 loop is a global property of PH domains. For the β-spectrin and ArhGap9 PH domains that bind PIP molecules at non-canonical (Ceccarelli et al., 2007;

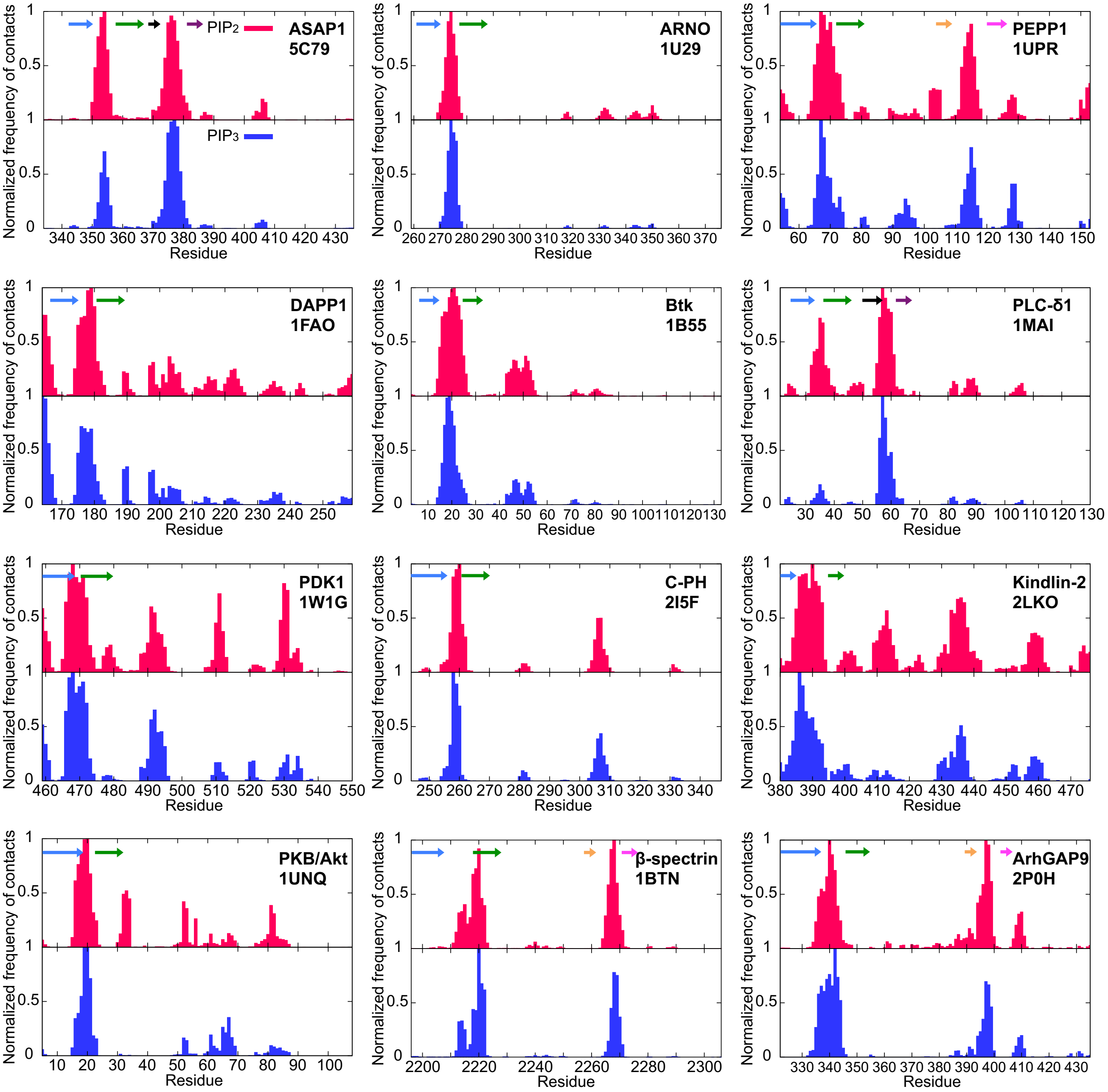

Figure 4. Normalized Average Number of Contacts between the PH Domains and PIPs

Contacts were calculated using the whole ensemble (25 × 1 μs CG-MD simulations). For normalization, the number of contacts of a residue with a lipid type was divided by the largest number of contacts that the same lipid type made with any residue in the PH domain. The positions of the β1 to β6 stands are shown by blue, green, black, purple, orange, and pink arrows, respectively. See also Figures S5 and S6.

Moravcevic et al., 2012), the secondary binding region is located in the β5/β6 loop.

DISCUSSION

In this study, we have shown that a molecular simulation protocol can yield structural data for PH/PIP complexes that are directly comparable to the complexes obtained from NMR and X-ray crystallography. This result is significant from both methodological and biological perspectives. Methodologically, we have shown that a high-throughput coarse-grained simulation approach, generating ensembles of simulations, can be used to study the structural and dynamic features of the association of PMPs with model membranes. This approach provides significant mechanistic details of the formation of the PH/bilayer complexes that are often difficult to obtain using experimental biophysical and structural techniques. Biologically, our results demonstrate that while the PH/PIP interaction occurs primarily

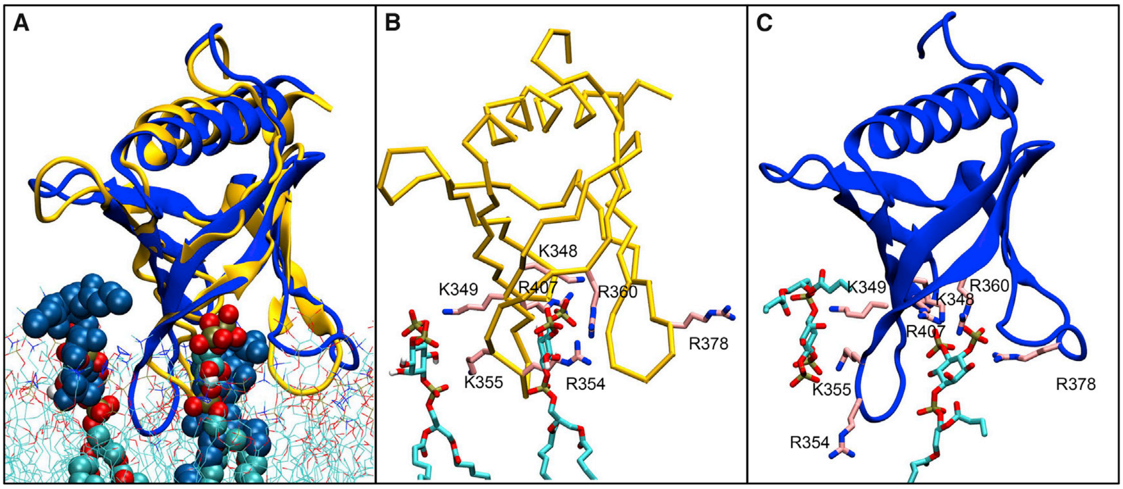

Figure 5. Binding of PIP Molecules to Both Canonical and Non-canonical Sites on the ASAP1 PH Domain

(A–C) A simulation snapshot and the crystal structure are compared in (A) (the same format as in Figure 3 for other PH domains). (B) A snapshot of the PH/PIP_2 complex derived from our CG simulations and then converted to an atomistic model, with the PH domain in yellow and the two bound PIP_2 molecules (in cyan/red/bronze). (C) The crystal structure (PDB: 5C79) with the PH domain in blue and the two bound dibutyryl PIP molecules (in cyan/red/bronze/silver). See also Figure S2.

via the β1/β2 loop region, in a number of PH domains a secondary (non-canonical) lipid-binding site is seen.

The major finding of our study is that the β1/β2 loop region constitutes the primary PIP-binding site on PH domains. This is in good agreement with experimental data on the GRP1 and ARNO PH domains, which suggest that mutations of the β1/β2 loop residues abolish/reduce the interactions of the aforementioned PH domains with PIP headgroups (Cronin et al., 2004). In particular, abolishment (K273A mutation) and reduction (R277A and K282A mutations) of PIP_3 binding with GRP1 are observed (Cronin et al., 2004). Mutations on the K173 residue on DAPP1 abolished the binding to 3-phosphoinositides (Dowler et al., 1999). Similarly, mutations of positive residues on the β1/β2 loop of the Bam32 PH domain (Marshall et al., 2000), PKB PH (Thomas et al., 2002), PDK1 PH (Anderson et al., 1998; Sundaresan et al., 2011), Kindlin 2 PH (Liu et al., 2011; Qu et al., 2011), and ArhGap9 PH (Ceccarelli et al., 2007) also resulted in the decrease/abolishment of their interactions with PIP molecules. In our CG-MD simulation with a K173L mutation on the DAPP1 PH domain, we observed a reduction in the interaction with PIP_2 and an increase in the probability of the PH domain adopting a different orientation on the membrane surface (see Figure S1). This, in combination with the fact that there are many positively charged amino acids (i.e., lysine and arginine) in the loop between β1 and β2 in all PH domains, suggests that this is a general property of lipid-binding PH domains (Carpten et al., 2007; He et al., 2011).

Importantly, our simulation approach is also able to identify secondary (non-canonical) lipid-binding sites on PH domains. In good agreement with a recent crystallographic study of the ASAP1 PH domain, we could identify two PIP-binding sites on the ASAP1 PH domain (Jian et al., 2015), while suggesting a more physiologically relevant orientation of PIP at the non-canonical binding site. Binding of PIP molecules to atypical (or non-canonical) lipid-binding sites has also been suggested for Sim1, Tiam, β-spectrirn, and ArhGap9 PH domains (Anand et al., 2012; Ceccarelli et al., 2007; Hyvönen et al., 1995; Moravcevic et al., 2012). A recent study on 91 yeast PH domains also showed that the presence of PIP lipids is required for the recruitment of PH domains to the membrane. However, the presence of other lipids often regulated their affinity and specificity (Vonkova et al., 2015). This provides evidence that multiple lipid interactions are crucial for the binding of PH domains to membranes. In addition to the PIP binding to secondary binding sites, we also observe a degree of clustering of PIP lipids around the PH domains. This clustering of PIP lipids may reorganize the local lipid environment creating PIP nanodomains. These PIP nanodomains may in turn be important for the clustering/recruitment of other peripheral or integral membrane proteins (van den Bogaart et al., 2011).

The use of a high-throughput methodology ensures effective sampling in the CG-MD simulations. Despite the known approximations of the CG methodology (discussed in, e.g., Marrink and Tieleman, 2013), the current study provides a paradigm for how a computational pipeline may be used to systematically study and quantify the interactions of multiple members of a family of membrane proteins. Our high-throughput approach makes it easy to simulate not only large number of proteins but also to explore effects of changes in the lipid environment. Given the ongoing increase in the available computational resources, this approach is readily scalable to all the structures of the PH domains for which there are structures in the PDB. It can also be easily extended to a wider range of PMPs, e.g., proteins containing C2 or FERM domains. Recently, similar pipelines have been developed for studying the oligomerization of TM helices (Wassenaar et al., 2015), the interaction of PIP lipids with human RTKs TM and juxtamembrane regions (Hedger et al., 2015), and the insertion of integral membrane proteins into bilayers (Stansfeld et al., 2015). This further proves the feasibility of membrane protein simulation pipelines, and

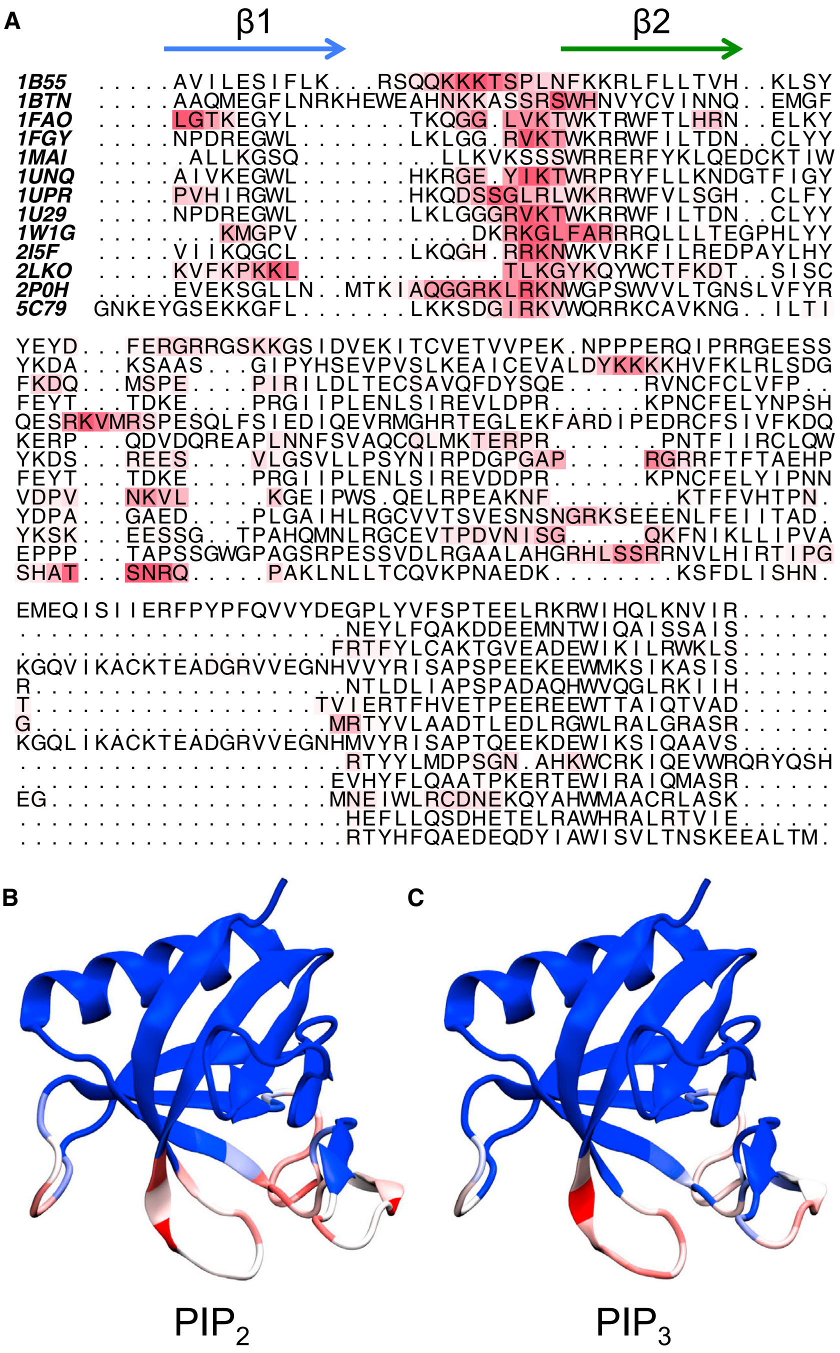

Figure 6. Conservation of the Interactions with PIP Lipids

(A–C) Sequence alignment of the PH domains used in this study (A). Red indicates a high number of contacts, whereas white indicate no contacts. Structures of the GRP1 PH domain color-coded based on the number of contact with PIP_2 (B) or PIP_3 (C) (both averaged over 25 × 1 μs CG-MD simulations of the GRP1). Blue indicates no contacts, and red a high number of contacts. See also Figure S6.

demonstrates that they can be extended to other a variety of membrane protein systems.

EXPERIMENTAL PROCEDURES

Coarse-Grained Molecular Dynamics Simulations

CG-MD simulations were performed using GROMACS-4.5.5 (Hess et al., 2008) (also see www.gromacs.org) with the Martini 2.1 force field (Marrink et al., 2007; Monticelli et al., 2008). The simulation systems are shown in Figure S1. The bilayer used in the simulations was comprised of 259 palmitoyloleoyl-phosphatidylcholine (POPC) (73%), 71 palmitoyloleoylglycerophosphoserine (POPS) (20%), 18 PIP_2 (5%), and 8 PIP_3 (2%) lipid molecules. Note that PIP_2 refers to PI(4,5)P_2 and PIP_3 refers to PI(3,4,5)P_3. Each leaflet thus contained 9 PIP_2 and 4 PIP_3 molecules. The systems were solvated with ~14,000 CG water molecules, and NaCl ions at 150 mM concentration were added to neutralize the system. Flexible loop regions missing from the PH structures and a mutation on the DAPP1 PH domain (K173L) were modeled using MODELLER (Fiser and Šali, 2003). All systems were energy minimized for 200 steps, and equilibrated for 1 ns with the protein backbone particle restrained. For each repeat simulation within an ensemble, the protein was rotated around the *x*, *y*, and *z* axes to form a different initial configuration. For each system an ensemble of 25 simulations of 1.0 μs each were run with a time step of 20 fs. An elastic network model was applied to all backbone particles with a cut-off distance of 0.7 nm to model secondary and tertiary structure (Atilgan et al., 2001). The bond length was constrained to equilibrium lengths using the linear constraint solver (LINCS) algorithm (for molecular simulations) (Hess et al., 1997). Lennard-Jones interactions were shifted to zero between 0.9 and 1.2 nm and Coulombic interactions between 0 and 1.2 nm, respectively. The pressure of 1 bar and temperature of 323 K were controlled using the Berendsen algorithm (Berendsen et al., 1984) with a coupling time of 1 ps.

Atomistic Molecular Dynamics Simulations

Conversion of CG to atomistic systems was made using a fragment-based approach (Stansfeld and Sansom, 2011a). We performed 2 × 1.0 μs MD simulations for each system of the GRP1 and ASAP1 PH domains and 1 μs MD simulations for each of the PLC-δ1 and β-spectrin PH domains. For the initial configurations, we picked up from 1 μs CG-MDs where the PH domain was in preferable orientation and had a bound PIP at the experimentally suggested binding site. The GROMOS96 43a1 force field (Scott et al., 1999) was used with simple point charge water molecules using GROMACS-4.5.5 software. The temperature of 323 K was controlled using the velocity rescaling method (Bussi et al., 2007) with a coupling time of 0.1 ps. The pressure of 1 bar was controlled with semi-isotropic pressure coupling using the Parrinello-Rahman barostat (Parrinello and Rahman, 1981) with a coupling time of 1 ps. Bond lengths were constrained to equilibrium lengths using the LINCS method. The time step was set at 2 fs. The particle mesh Ewald method was used, with a specified direct space cut-off distance of 1.0 nm.

Density Maps of the Orientation of the PH Domains Relative to the Lipid Membrane

To investigate orientation of the PH domains relative to the lipid membrane, we consider the density map of the PH domains. Here, we have calculated the 2D

normalized histogram of R_{zz} and d_z, where d_z is the perpendicular distance between the centers of mass of a PH domain and the lipid membrane, and where R_{zz} is the *zz* component of the rotational matrix required for least squares fitting of a protein orientation onto a reference orientation. R_{zz} was calculated using the *g_rotmat* command in GROMACS. The value of R_{zz} in the density map varies depending on the reference orientation of the PH domain relative to the membrane. The change in the normalized density map of system can be calculated from

$$\Delta D(R_{zz},d_z) = \rho(R_{zz},d_z)/\rho_0,$$

where $\rho(R_{zz},d_z)$ and ρ_0 are probabilities at a bin (R_{zz},d_z) and a reference point (which corresponds to the global maximum), respectively. Note that prior to the calculation, the rotation and translation of the protein in the *xy* plane was fitted using the *trjconv* command in GROMACS (Hess et al., 2008). The ensemble used for the calculation is 25 × 1.0 μs for CG-MD, 2 × 1.0 μs for AT-MD of GRP1 and ASAP1, and 1 × 1.0 μs for AT-MD of PLC-δ1 and β-spectrin. Similar methodologies were previously used to calculate the energy landscapes of single-tail lipid flip-flop (Arai et al., 2014) and of the insertion of hydrophobic peptides to model membrane (Ulmschneider et al., 2011).

SUPPLEMENTAL INFORMATION

Supplemental Information includes seven figures and can be found with this article online at http://dx.doi.org/10.1016/j.str.2016.06.002.

AUTHOR CONTRIBUTIONS

E.Y. and A.C.K. performed the calculation and analysis. The research reported here emerged from lively discussions between E.Y., A.C.K., K.Y., and M.S.P.S. All authors contributed to writing the manuscript.

ACKNOWLEDGMENTS

This work was supported by MEXT Grant-in-Aid for the Program for Leading Graduate Schools and Keio University Doctorate Student Grant-in-Aid Program. A.C.K. and M.S.P.S. were funded by the Wellcome Trust. We acknowledge the EPSRC and the UK High-End Computing Consortium for Biomolecular Simulation for providing computational time on the ARCHER supercomputer.

Received: January 21, 2016
Revised: May 10, 2016
Accepted: June 2, 2016
Published: July 14, 2016

REFERENCES

Anand, K., Maeda, K., and Gavin, A.-C. (2012). Structural analyses of the Slm1-PH domain demonstrate ligand binding in the non-canonical site. PLoS One *7*, e36526.

Anderson, K.E., Coadwell, J., Stephens, L.R., and Hawkins, P.T. (1998). Translocation of PDK-1 to the plasma membrane is important in allowing PDK-1 to activate protein kinase B. Curr. Biol. *8*, 684–691.

Arai, N., Akimoto, T., Yamamoto, E., Yasui, M., and Yasuoka, K. (2014). Poisson property of the occurrence of flip-flops in a model membrane. J. Chem. Phys. *140*, 064901.

Atilgan, A.R., Durell, S.R., Jernigan, R.L., Demirel, M.C., Keskin, O., and Bahar, I. (2001). Anisotropy of fluctuation dynamics of proteins with an elastic network model. Biophys. J. *80*, 505–515.

Balla, T. (2005). Inositol-lipid binding motifs: signal integrators through protein-lipid and protein-protein interactions. J. Cell Sci. *118*, 2093–2104.

Baraldi, E., Carugo, K.D., Hyvönen, M., Surdo, P.L., Riley, A.M., Potter, B.V.L., O'Brien, R., Ladbury, J.E., and Saraste, M. (1999). Structure of the PH domain from Bruton's tyrosine kinase in complex with inositol 1,3,4,5-tetrakisphosphate. Structure *7*, 449–460.

Berendsen, H.J.C., Postma, J.P.M., van Gunsteren, W.F., DiNola, A., and Haak, J.R. (1984). Molecular dynamics with coupling to an external bath. J. Chem. Phys. *81*, 3684–3690.

Best, M.D. (2014). Global approaches for the elucidation of phosphoinositide-binding proteins. Chem. Phys. Lipids *182*, 19–28.

Bussi, G., Donadio, D., and Parrinello, M. (2007). Canonical sampling through velocity rescaling. J. Chem. Phys. *126*, 014101.

Carpten, J.D., Faber, A.L., Horn, C., Donoho, G.P., Briggs, S.L., Robbins, C.M., Hostetter, G., Boguslawski, S., Moses, T.Y., Savage, S., et al. (2007). A transforming mutation in the pleckstrin homology domain of AKT1 in cancer. Nature *448*, 439–444.

Ceccarelli, D.F.J., Blasutig, I.M., Goudreault, M., Li, Z., Ruston, J., Pawson, T., and Sicheri, F. (2007). Non-canonical interaction of phosphoinositides with pleckstrin homology domains of Tiam1 and ArhGAP9. J. Biol. Chem. *282*, 13864–13874.

Chen, H.-C., Ziemba, B.P., Landgraf, K.E., Corbin, J.A., and Falke, J.J. (2012). Membrane docking geometry of GRP1 PH domain bound to a target lipid bilayer: an EPR site-directed spin-labeling and relaxation study. PLoS One *7*, e33640.

Cho, W., and Stahelin, R.V. (2005). Membrane-protein interactions in cell signaling and membrane trafficking. Annu. Rev. Biophys. Biomol. Struct. *34*, 119–151.

Corbin, J.A., Dirkx, R.A., and Falke, J.J. (2004). GRP1 Pleckstrin homology domain: activation parameters and novel search mechanism for rare target lipid. Biochemistry *43*, 16161–16173.

Cronin, T.C., DiNitto, J.P., Czech, M.P., and Lambright, D.G. (2004). Structural determinants of phosphoinositide selectivity in splice variants of Grp1 family PH domains. EMBO J. *23*, 3711–3720.

Dowler, S., Currie, R.A., Downes, C.P., and Alessi, D.R. (1999). DAPP1: a dual adaptor for phosphotyrosine and 3-phosphoinositides. Biochem. J. *342*, 7–12.

Ferguson, K.M., Lemmon, M.A., Schlessinger, J., and Sigler, P.B. (1995). Structure of the high affinity complex of inositol trisphosphate with a phospholipase C pleckstrin homology domain. Cell *83*, 1037–1046.

Ferguson, K.M., Kavran, J.M., Sankaran, V.G., Fournier, E., Isakoff, S.J., Skolnik, E.Y., and Lemmon, M.A. (2000). Structural basis for discrimination of 3-Phosphoinositides by pleckstrin homology domains. Mol. Cell *6*, 373–384.

Fiser, A., and Šali, A. (2003). Modeller: generation and refinement of homology-based protein structure models. Methods Enzymol. *374*, 461–491.

Hancock, J.F. (2003). Ras proteins: different signals from different locations. Nat. Rev. Mol. Cell Biol. *4*, 373–385.

He, J., Scott, J.L., Heroux, A., Roy, S., Lenoir, M., Overduin, M., Stahelin, R.V., and Kutateladze, T.G. (2011). Molecular basis of phosphatidylinositol 4-phosphate and ARF1 GTPase recognition by the FAPP1 pleckstrin homology (PH) domain. J. Biol. Chem. *286*, 18650–18657.

Hedger, G., Sansom, M.S.P., and Koldso, H. (2015). The juxtamembrane regions of human receptor tyrosine kinases exhibit conserved interaction sites with anionic lipids. Sci. Rep. *5*, 9198.

Hess, B., Bekker, H., Berendsen, H., and Fraaije, J. (1997). LINCS: a linear constraint solver for molecular simulations. J. Comput. Chem. *18*, 1463–1472.

Hess, B., Kutzner, C., van der Spoel, D., and Lindahl, E. (2008). GROMACS 4: algorithms for highly efficient, load-balanced, and scalable molecular simulation. J. Chem. Theory Comput. *4*, 435–447.

Huang, B.X., Akbar, M., Kevala, K., and Kim, H.-Y. (2011). Phosphatidylserine is a critical modulator for Akt activation. J. Cell Biol. *192*, 979–992.

Hyvönen, M., Macias, M.J., Nilges, M., Oschkinat, H., Saraste, M., and Wilmanns, M. (1995). Structure of the binding site for inositol phosphates in a PH domain. EMBO J. *14*, 4676–4685.

Jackson, S., Zhang, Y., Haslam, R., and Junop, M. (2007). Structural analysis of the carboxy terminal PH domain of pleckstrin bound to D-myo-inositol 1,2,3,5,6-pentakisphosphate. BMC Struct. Biol. *7*, 80.

Jian, X., Tang, W.-K., Zhai, P., Roy, N.S., Luo, R., Gruschus, J.M., Yohe, M.E., Chen, P.-W., Li, Y., Byrd, R.A., et al. (2015). Molecular basis for cooperative

binding of anionic phospholipids to the PH domain of the Arf GAP ASAP1. Structure *23*, 1977–1988.

Kalli, A.C., Devaney, I., and Sansom, M.S.P. (2014). Interactions of phosphatase and tensin homologue (PTEN) proteins with phosphatidylinositol phosphates: insights from molecular dynamics simulations of PTEN and voltage sensitive phosphatase. Biochemistry *53*, 1724–1732.

Kalli, A.C., and Sansom, M.S.P. (2014). Interactions of peripheral proteins with model membranes as viewed by molecular dynamics simulations. Biochem. Soc. Trans. *42*, 1418–1424.

Kavran, J.M., Klein, D.E., Lee, A., Falasca, M., Isakoff, S.J., Skolnik, E.Y., and Lemmon, M.A. (1998). Specificity and promiscuity in phosphoinositide binding by pleckstrin homology domains. J. Biol. Chem. *273*, 30497–30508.

Klarlund, J.K., Tsiaras, W., Holik, J.J., Chawla, A., and Czech, M.P. (2000). Distinct polyphosphoinositide binding selectivities for pleckstrin homology domains of GRP1-like proteins based on diglycine versus triglycine motifs. J. Biol. Chem. *275*, 32816–32821.

Knight, J.D., Lerner, M.G., Marcano-Velázquez, J.G., Pastor, R.W., and Falke, J.J. (2010). Single molecule diffusion of membrane-bound proteins: window into lipid contacts and bilayer dynamics. Biophys. J. *99*, 2879–2887.

Komander, D., Fairservice, A., Deak, M., Kular, G.S., Prescott, A.R., Peter Downes, C., Safrany, S.T., Alessi, D.R., and van Aalten, D.M.F. (2004). Structural insights into the regulation of PDK1 by phosphoinositides and inositol phosphates. EMBO J. *23*, 3918–3928.

Kutateladze, T., and Overduin, M. (2001). Structural mechanism of endosome docking by the FYVE domain. Science *291*, 1793–1796.

Lai, C.-L., Srivastava, A., Pilling, C., Chase, A.R., Falke, J.J., and Voth, G.A. (2013). Molecular mechanism of membrane binding of the GRP1 PH domain. J. Mol. Biol. *425*, 3073–3090.

Lemmon, M.A. (2008). Membrane recognition by phospholipid-binding domains. Nat. Rev. Mol. Cell Biol. *9*, 99–111.

Lietzke, S.E., Bose, S., Cronin, T., Klarlund, J., Chawla, A., Czech, M.P., and Lambright, D.G. (2000). Structural basis of 3-phosphoinositide recognition by peckstrin homology domains. Mol. Cell *6*, 385–394.

Liu, J., Fukuda, K., Xu, Z., Ma, Y.-Q., Hirbawi, J., Mao, X., Wu, C., Plow, E.F., and Qin, J. (2011). Structural basis of phosphoinositide binding to kindlin-2 protein pleckstrin homology domain in regulating integrin activation. J. Biol. Chem. *286*, 43334–43342.

Lucas, N., and Cho, W. (2011). Phosphatidylserine binding is essential for plasma membrane recruitment and signaling function of 3-phosphoinositide-dependent kinase-1. J. Biol. Chem. *286*, 41265–41272.

Lumb, C.N., He, J., Xue, Y., Stansfeld, P.J., Stahelin, R.V., Kutateladze, T.G., and Sansom, M.S.P. (2011). Biophysical and computational studies of membrane penetration by the GRP1 pleckstrin homology domain. Structure *19*, 1338–1346.

Marrink, S.J., and Tieleman, D.P. (2013). Perspective on the Martini model. Chem. Soc. Rev. *42*, 6801–6822.

Marrink, S.J., Risselada, H.J., Yefimov, S., Tieleman, D.P., and de Vries, A.H. (2007). The MARTINI force field: coarse grained model for biomolecular simulations. J. Phys. Chem. B *111*, 7812–7824.

Marshall, A.J., Niiro, H., Lerner, C.G., Yun, T.J., Thomas, S., Disteche, C.M., and Clark, E.A. (2000). A novel B lymphocyte-associated adaptor protein, Bam32, regulates antigen receptor signaling downstream of phosphatidylinositol 3-kinase. J. Exp. Med. *191*, 1319–1332.

Milburn, C.C., Deak, M., Kelly, S.M., Price, N.C., Alessi, D.R., and Van Aalten, D.M.F. (2003). Binding of phosphatidylinositol 3,4,5-trisphosphate to the pleckstrin homology domain of protein kinase B induces a conformational change. Biochem. J. *375*, 531–538.

Monticelli, L., Kandasamy, S.K., Periole, X., Larson, R.G., Tieleman, D.P., and Marrink, S.-J. (2008). The MARTINI coarse-grained force field: extension to proteins. J. Chem. Theory Comput. *4*, 819–834.

Moravcevic, K., Oxley, C.L., and Lemmon, M.A. (2012). Conditional peripheral membrane proteins: facing up to limited specificity. Structure *20*, 15–27.

Naughton, F.B., Kalli, A.C., and Sansom, M.S.P. (2016). Association of peripheral membrane proteins with membranes: free energy of binding of GRP1 PH domain with PIP-containing model bilayers. J. Phys. Chem. Lett. *7*, 1219–1224.

Parrinello, M., and Rahman, A. (1981). Polymorphic transitions in single crystals: a new molecular dynamics method. J. Appl. Phys. *52*, 7182–7190.

Picas, L., Viaud, J., Schauer, K., Vanni, S., Hnia, K., Fraisier, V., Roux, A., Bassereau, P., Gaits-Iacovoni, F., Payrastre, B., et al. (2014). BIN1/M-Amphiphysin2 induces clustering of phosphoinositides to recruit its downstream partner dynamin. Nat. Commun. *5*, 5647.

Qu, H., Tu, Y., Shi, X., Larjava, H., Saleem, M.A., Shattil, S.J., Fukuda, K., Qin, J., Kretzler, M., and Wu, C. (2011). Kindlin-2 regulates podocyte adhesion and fibronectin matrix deposition through interactions with phosphoinositides and integrins. J. Cell Sci. *124*, 879–891.

Scott, W.R.P., Hunenberger, P.H., Tironi, I.G., Mark, A.E., Billeter, S.R., Fennen, J., Torda, A.E., Huber, T., Kruger, P., and van Gunsteren, W.F. (1999). The GROMOS biomolecular simulation program package. J. Phys. Chem. A *103*, 3596–3607.

Stahelin, R.V., Scott, J.L., and Frick, C.T. (2014). Cellular and molecular interactions of phosphoinositides and peripheral proteins. Chem. Phys. Lipids *182*, 3–18.

Stansfeld, P.J., and Sansom, M.S.P. (2011a). From coarse grained to atomistic: a serial multiscale approach to membrane protein simulations. J. Chem. Theory Comput. *7*, 1157–1166.

Stansfeld, P.J., and Sansom, M.S.P. (2011b). Molecular simulation approaches to membrane proteins. Structure *19*, 1562–1572.

Stansfeld, P.J., Goose, J.E., Caffrey, M., Carpenter, E.P., Parker, J.L., Newstead, S., and Sansom, M.S.P. (2015). MemProtMD: automated insertion of membrane protein structures into explicit lipid membranes. Structure *23*, 1350–1361.

Sundaresan, N.R., Pillai, V.B., Wolfgeher, D., Samant, S., Vasudevan, P., Parekh, V., Raghuraman, H., Cunningham, J.M., Gupta, M., and Gupta, M.P. (2011). The deacetylase SIRT1 promotes membrane localization and activation of Akt and PDK1 during tumorigenesis and cardiac hypertrophy. Sci. Signal *4*, ra46.

Thomas, C.C., Deak, M., Alessi, D.R., and van Aalten, D.M.F. (2002). High-resolution structure of the pleckstrin homology domain of protein kinase B/Akt bound to phosphatidylinositol (3,4,5)-trisphosphate. Curr. Biol. *12*, 1256–1262.

Ulmschneider, J.P., Smith, J.C., White, S.H., and Ulmschneider, M.B. (2011). In silico partitioning and transmembrane insertion of hydrophobic peptides under equilibrium conditions. J. Am. Chem. Soc. *133*, 15487–15495.

van den Bogaart, G., Meyenberg, K., Risselada, H.J., Amin, H., Willig, K.I., Hubrich, B.E., Dier, M., Hell, S.W., Grubmuller, H., Diederichsen, U., et al. (2011). Membrane protein sequestering by ionic protein-lipid interactions. Nature *479*, 552–555.

Vermaas, J., Baylon, J., Arcario, M., Muller, M., Wu, Z., Pogorelov, T., and Tajkhorshid, E. (2015). Efficient exploration of membrane-associated phenomena at atomic resolution. J. Membr. Biol. *248*, 563–582.

Vonkova, I., Saliba, A.-E., Deghou, S., Anand, K., Ceschia, S., Doerks, T., Galih, A., Kugler, K.G., Maeda, K., Rybin, V., et al. (2015). Lipid cooperativity as a general membrane-recruitment principle for PH domains. Cell Rep. *12*, 1519–1530.

Wassenaar, T.A., Pluhackova, K., Moussatova, A., Sengupta, D., Marrink, S.J., Tieleman, D.P., and Bockmann, R.A. (2015). High-throughput simulations of dimer and trimer assembly of membrane proteins. The DAFT approach. J. Chem. Theory Comput. *11*, 2278–2291.

Yamamoto, E., Kalli, A.C., Akimoto, T., Yasuoka, K., and Sansom, M.S.P. (2015). Anomalous dynamics of a lipid recognition protein on a membrane surface. Sci. Rep. *5*, 18245.

Structure, Volume *24*

Supplemental Information

Interactions of Pleckstrin Homology Domains with Membranes: Adding Back the Bilayer via High-Throughput Molecular Dynamics

Eiji Yamamoto, Antreas C. Kalli, Kenji Yasuoka, and Mark S.P. Sansom

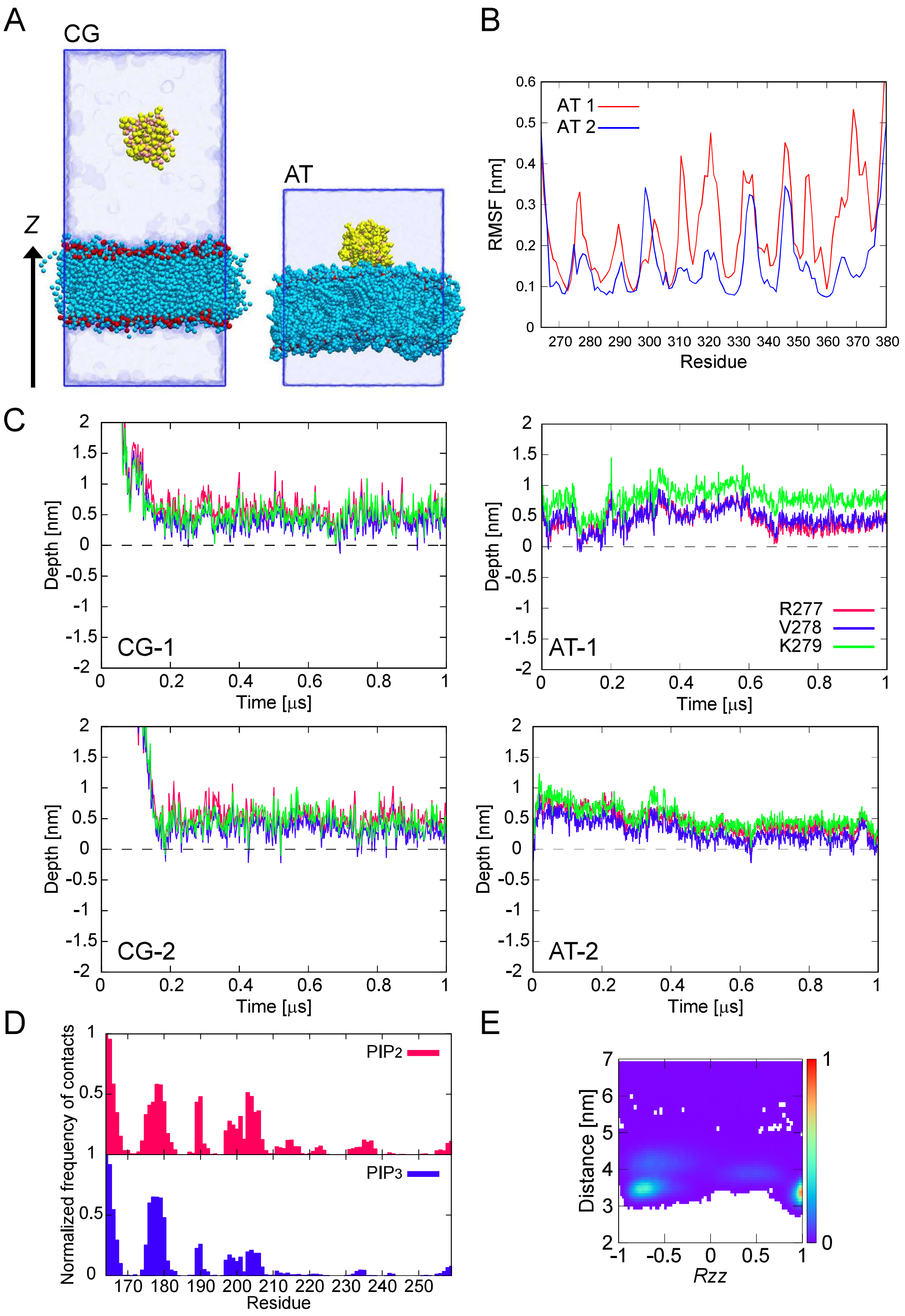

Figure S1, related to Figures 1 and 2. PH domain/bilayer simulations and analysis.
(A) The initial system used for MD simulations. The PH domain and lipid bilayer are shown in yellow (Cα atom: pink) and light blue (phosphate particle: red), respectively. Water molecules are indicated by the upper and lower transparent coatings. The blue line of the box defines the unit cell. The system sizes $L_x \times L_y \times L_z$ are CG: 10.9×10.9×20.1 nm^3 and AT: 10.5×10.5×12.9 nm^3. (B) Root Mean Square Fluctuation (RMSF) analysis for GRP1 PH domain for the AT-MD simulations. The

RMSF values were calculated from the last 0.9 μs of AT-MD trajectories. The first 0.1 μs were discarded as "equilibration" periods. (C) Penetration of GRP1 PH domain into the membrane. The depths of the Cα atoms of R277, V278, and K279 residues with respect to the membrane surface (defined by the average position of phosphates of the lipids, shown as a dashed line at 0) were calculated. (D,E) Mutation in the DAPP1 PH domain (K173L). (D) Normalized average number of contacts between the PH domain and PIP molecules are shown for the 25×1 μs CG-MD simulations. The contact of residues around 164-166 and 176-180 with PIP_2 are changed by this mutation (see Fig. 4 in main text). (E) Normalized density map for the PH domain (*zz* component of rotational matrix *vs* distance). The state around Rzz = -0.8 increased by this mutation compared with that of the WT (see Fig. 3 in main text).

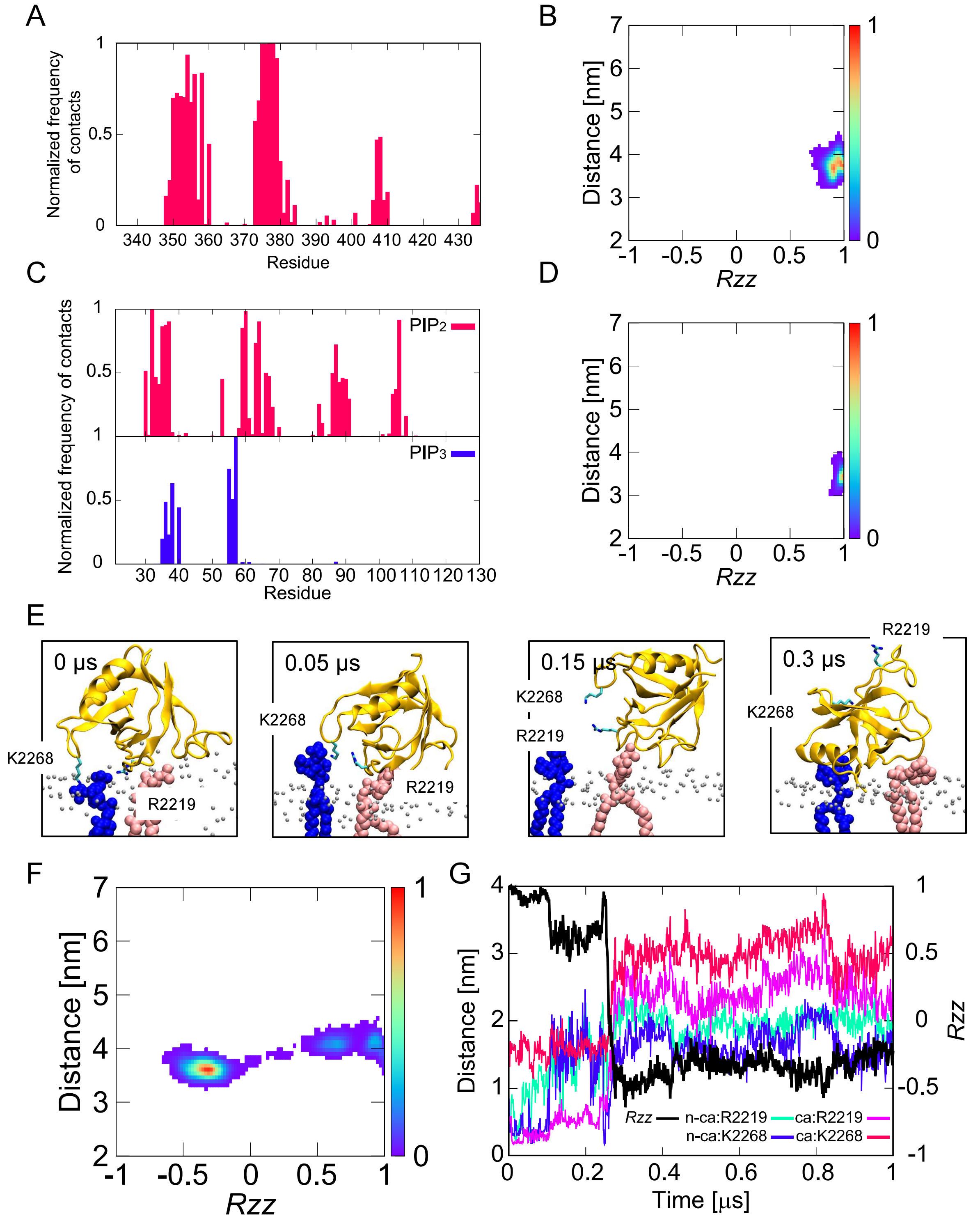

Figure S2, related to Figures 1 and 5. Atomistic simulations of PH domains

(A,B) AT-MD of the ASAP1 PH domain. (A) Normalized average number of contacts between the PH domain and PIP_2 (cutoff distance 0.4 nm) shown for the 2×1 μs AT-MD simulations. (B) Normalized density map for the PH domain (*zz* component of rotational matrix *vs* distance). (C,D) AT-MD of the PLC-δ1 PH domain. (C) Normalized average number of contacts between the PH domain and PIPs (cutoff distance 0.4 nm) are shown for the 1 μs AT-MD simulation. (D) Normalized density map for the PH domain (*zz* component of rotational matrix *vs* distance). (E-G)

AT-MD of the β-spectrin PH domain. (E) Snapshots of one of the atomistic simulations with the PH domain (yellow) with PIPs in the non-canonical (blue) and canonical (pink) sites. The phosphate atoms of lipids are coloured in silver. (F) Normalized density map for the PH domain (*zz* component of rotational matrix *vs* distance) for the 1 μs AT-MD simulation. (G) R_{zz} (black solid line) and minimum distances between PIPs and the amino acid (R2219 and K2268) relative to the simulation time: green and blue lines show PIPs that are in the non-canonical site and pink and red lines show PIPs that are in the canonical site. When the PIP unbinds from the non-canonical site, the orientation of the PH domain changes to a different orientation. Interestingly, this orientation corresponds to a secondary orientation that we observe in the CG-MD simulation with the β-spectrin PH domain (see Fig. 3 in the main text). This suggests that our AT-MD simulation is long enough to see the different membrane-bound states (at the atomistic resolution) that we observe in the CG-MD simulations.

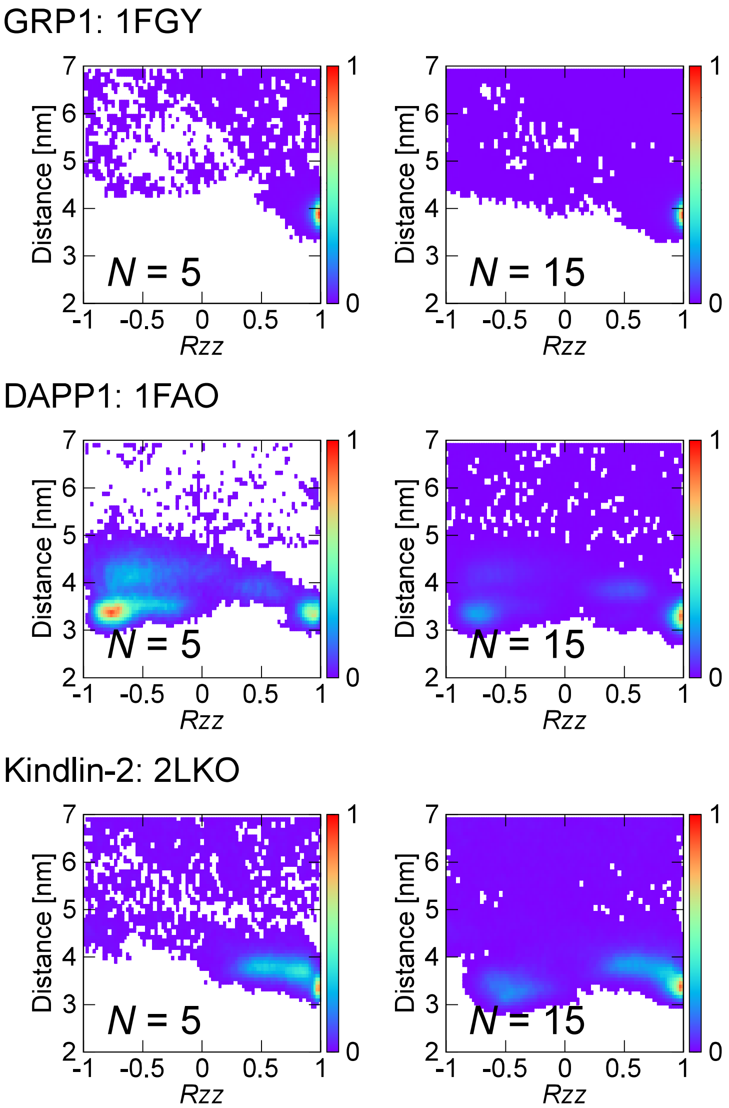

Figure S3, related to Figure 2. Convergence analysis of normalized density maps

The *zz* component of the rotational matrix (*Rzz*) *vs* the distance between centres of mass of the PH domain and the bilayer for the GRP1, DAPP1, and Kindlin-2 PH domains is shown for the ensemble when we have used 5 and 15 repeat simulations to construct the landscape. Those with 25 ensembles are shown in main text (see Fig. 3). This analysis suggests that the density landscape of the 25 ensembles was converged.

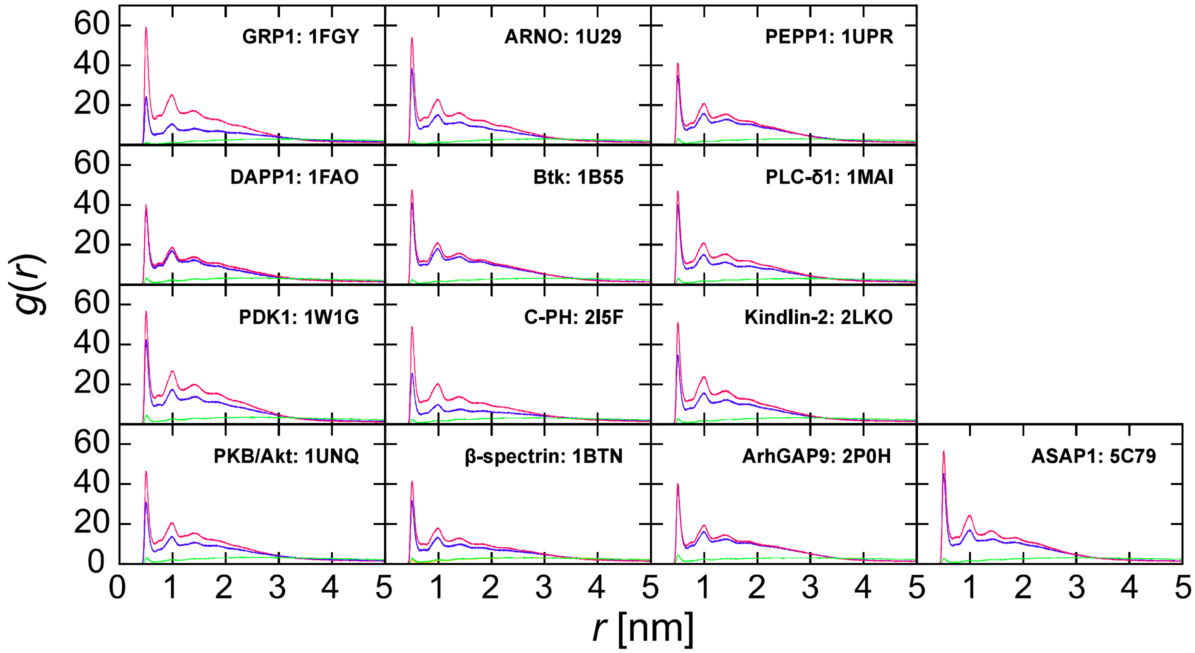

Figure S4, related to Figure 4. Clustering of lipid molecules around the PH domains

Radial distribution function $g(r)$ of lipid molecules around the PH domains derived from the CG-MD simulations. Red, blue, and green coloured lines represent the distribution of PIP_2, PIP_3, and POPS, respectively.

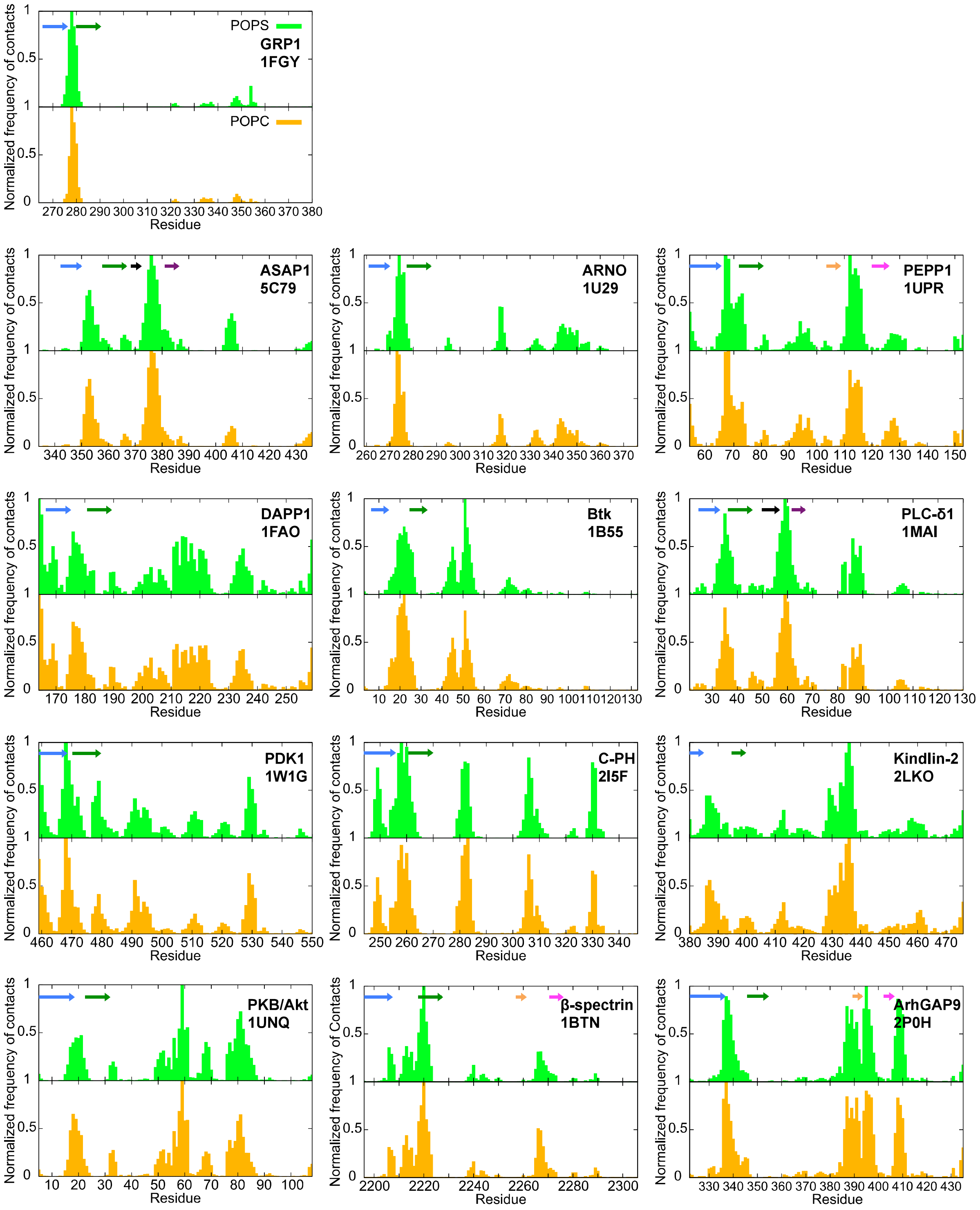

Figure S5, related to Figure 4. Normalized average number of contacts between PH domains and bilayer lipids

Contacts are shown for POPS (green) and POPC (yellow), respectively. The data are averaged over 25×1 μs CG-MD simulations.

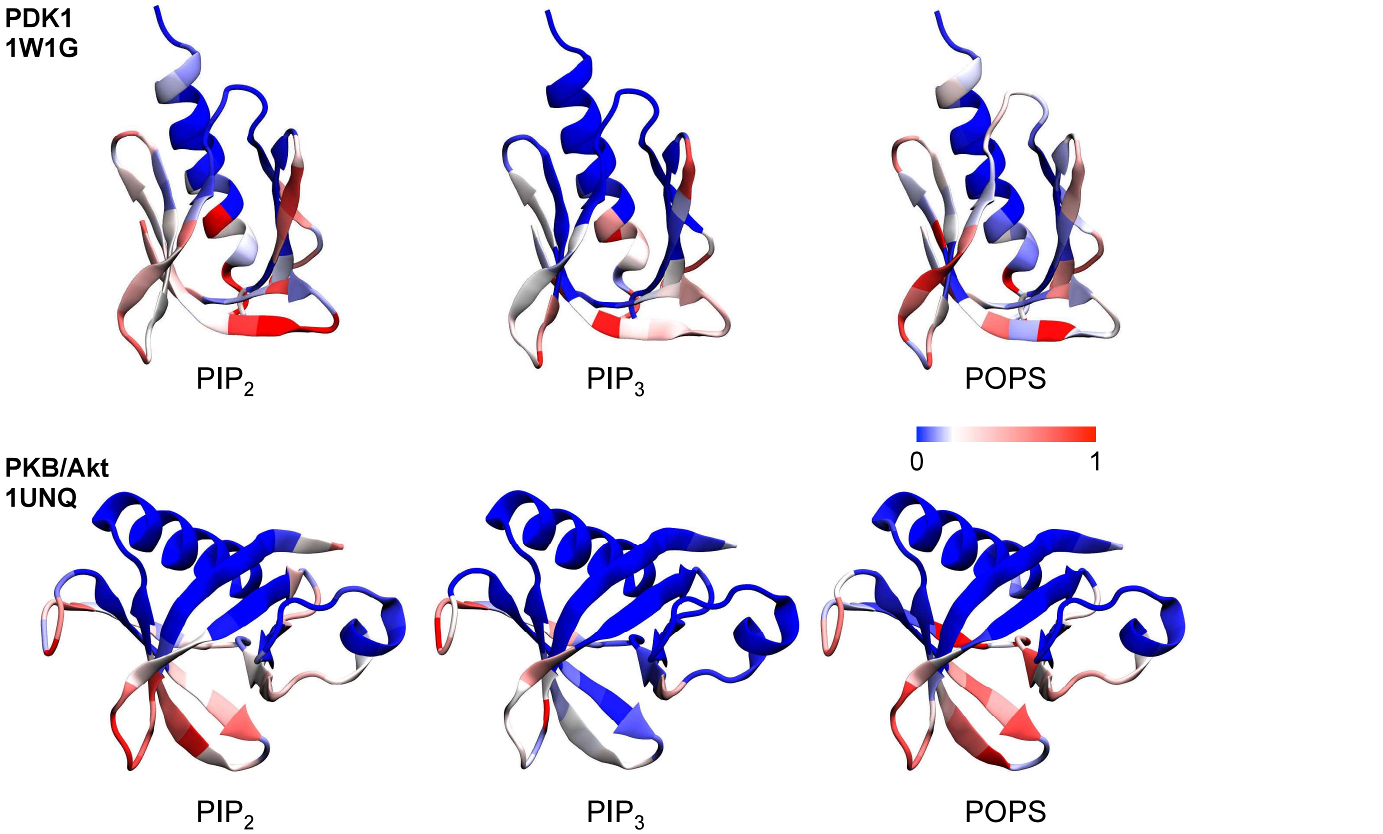

Figure S6, related to Figure 6. Contacts between PH domains and lipids

The amino acids are color-coded based on the normalized average number of contacts with the PIP_2, PIP_3, and POPS. Blue indicates a low number of contacts (or no contacts) and red a high number of contacts.

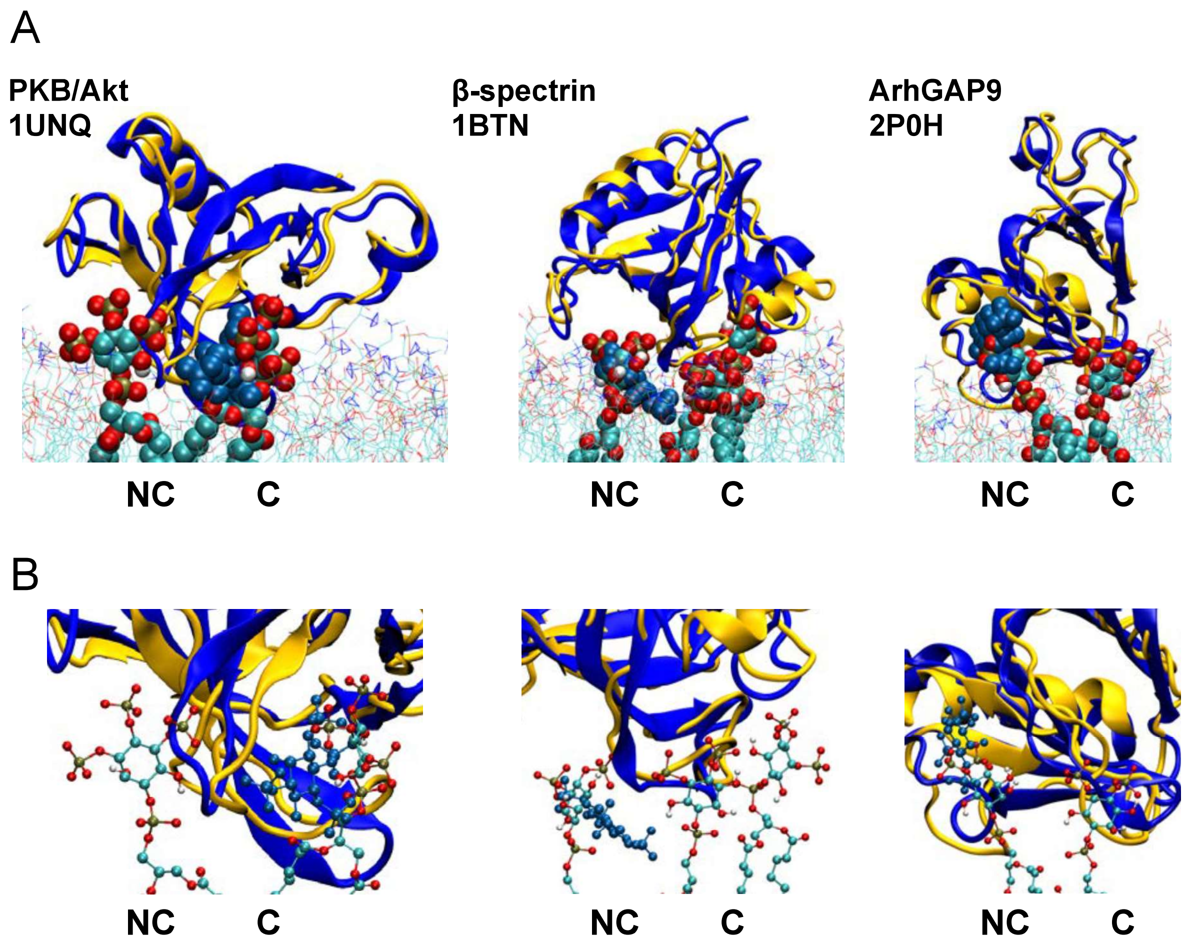

Figure S7, related to Figure 3. PIP lipids at canonical (C) and non-canonical (NC) binding sites

Alignment of the PH/PIP complex derived from our simulation approach (PH domain in yellow and PIP in cyan/red/bronze) with the crystal structure (PH domain and PIP both in blue). The figures in **B** show zoomed in versions of the figures in **A**, focussing on the bound PIP molecules.
